# Two decades of progress in gastric cancer peritoneal metastasis: a bibliometric perspective on molecular mechanisms and therapeutic innovations

**DOI:** 10.3389/fonc.2025.1583364

**Published:** 2025-05-30

**Authors:** Jin Qian, Xiaoxuan Tu, Jianglin Chen, Sisi Chen, Yi Zhou, Chuan Sun, Zhibing Wu

**Affiliations:** ^1^ Second Clinical Medical College, Zhejiang Chinese Medical University, Hangzhou, China; ^2^ Department of Oncology, Zhejiang Hospital, Hangzhou, China; ^3^ Zhejiang Key Laboratory of Geriatrics and Geriatrics Institute of Zhejiang Province, Zhejiang Hospital, Hangzhou, China; ^4^ Department of Radiation Oncology, Zhejiang Hospital, Hangzhou, China; ^5^ Department of Oncology, Affiliated Zhejiang Hospital, Zhejiang University School of Medicine, Hangzhou, China

**Keywords:** bibliometric, gastric cancer, peritoneal metastasis, molecular mechanisms, intraperitoneal treatment

## Abstract

**Background:**

Gastric cancer (GC) is the fifth most common malignant tumor worldwide. The peritoneum is a common site of metastasis in advanced GC, and patients with gastric cancer peritoneal metastasis (GCPM) have a very low 5-year survival rate. Systemic therapy has limited efficacy for peritoneal metastases, and early diagnosis is difficult. In this paper, we analyzed the GCPM-related literature by bibliometric methods, aiming to identify the research hotspots and trends and to provide a basis for clinical practice and research planning.

**Methods:**

Based on the Web of Science Core Collection database (WoSCC), we screened the GCPM-related literature published from 2004 to 2024. Countries, institutions, authors, journals, and keywords were analyzed and visualized by tools such as CiteSpace, VOSviewer, Scimago Graphica, RStudio, and the Bibliometrix package.

**Result:**

A total of 2416 publications were included in this study. The growth rate of GCPM publications is positive until 2021, with a slowdown in the near future. Japan dominated the research output (842 publications), followed by China (748 publications) and the United States (268 publications). Japanese-affiliated organizations and researchers are extremely productive in the field of GCPM. The most frequently cited document was Japanese gastric cancer treatment guidelines 2014 (ver. 4) (citations = 2076). Research focuses on four major clusters: (1) molecular mechanisms of GCPM; (2) prognosis of GCPM; (3) chemotherapy of GCPM; and (4) intraperitoneal treatment of GCPM. Emerging trends include key pathways of GCPM, artificial intelligence (AI) and multi-omics-driven early diagnosis, novel intraperitoneal therapeutic modalities, and immunologic/targeted drugs.

**Conclusion:**

Japan is a leader in GCPM research. Recently, the focus of GCPM research has shifted from basic treatment to precision and personalized treatment through the integration of molecular mechanisms, novel intraperitoneal therapeutic modalities, and AI technologies. Current challenges include the lack of standardized validation systems for emerging technologies and regional differences in clinical practice. In the future, there is a need to promote global collaborative trials and optimization of multimodality therapy. The results of this study provide a key direction and systematic basis for future exploration of GCPM.

## 1 Introduction

GC is the fifth most common malignant tumor and cancer-related cause of death worldwide (1). According to statistics, in 2022, there were about 968,000 new cases of GC and 660,000 deaths worldwide (2). Most patients are already in the progressive stage at the time of initial diagnosis. According to epidemiologic data from the United States, the Netherlands, and other countries, approximately 39% of GC patients are found to have distant metastasis at the time of initial diagnosis, and common sites of metastasis include the peritoneum, the liver, and distal lymph nodes, with approximately 40% of them being peritoneal metastases (3–6). Once metastasis occurs, the prognosis for metastatic GC is usually poor, with a median survival of only 3–6 months and a 5-year survival rate of less than 2% in patients who develop GCPM (5, 7–9). Currently, the main treatments for GC include surgery, chemotherapy, targeted therapy, and immunotherapy (10–12), but the treatment of GCPM is based only on systemic chemotherapy and supportive care and has a low early detection rate, leading to a poor prognosis for patients. A comprehensive understanding of the pathogenesis, therapeutic modalities, and prognostic factors of GCPM may be critical to improving the survival of patients with it.

Bibliometrics, a discipline introduced by Alan Pritchard in 1969, centers on the systematic analysis of scholarly publications to reveal patterns, trends, and impact in scientific research. After decades of development, bibliometric analysis has been widely used in the medical field (13). Researchers can use bibliometrics to analyze countries, institutions, authors, journals, and keywords in related fields and quantify and explain the spatial and temporal distribution of scholarly publications, collaborative networks, and keyword evolution with visualization tools, which can help researchers to systematically identify trends and research progress in related diseases and predict potential research hotspots, which is difficult to achieve with traditional reviews. There is a lack of comprehensive bibliometric articles to analyze the research trends and progress in the field of GCPM, in order to make up for the gap in this area, this study used bibliometric methods to study the GCPM-related literature from 2004-2024, to analyze and visualize the current status of research and treatment progress in the field of GCPM through quantitative indexes, and to predict the future hotspots of research in this field, in order to provide a reference and scientific research basis for the subsequent researchers to provide reference and scientific research basis.

## 2 Materials and methods

### 2.1 Search strategy

**Figure 1:**
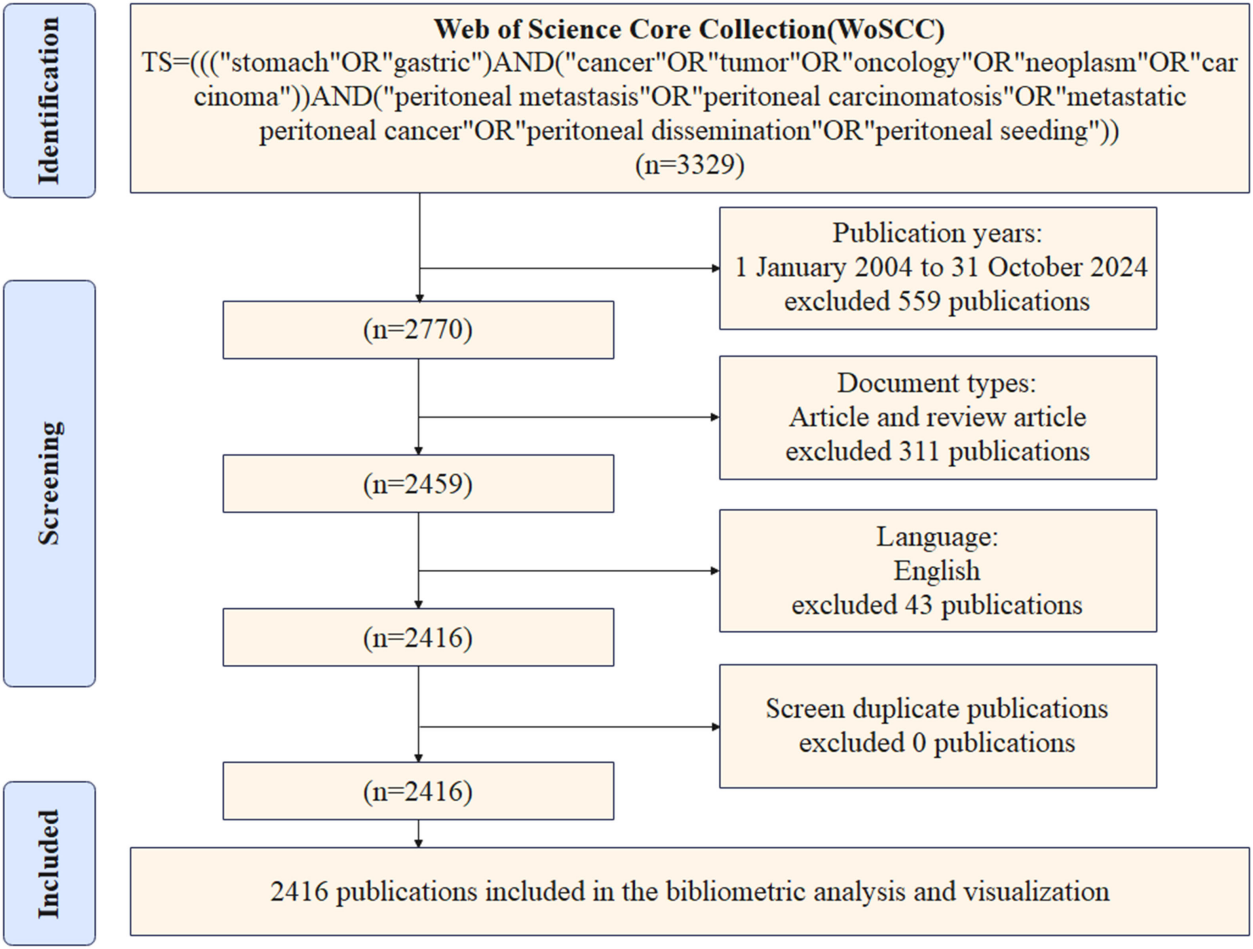
Literature search strategy for GCPM fields based on PRISMA 2020 guidelines. “Identification” shows the screening field, “Screening” shows the screening criteria, and “Included” shows the final included literature.

The literature included in this study was retrieved from the WoSCC database. A comparison of WoSCC and other databases such as PubMed, Scopus, Embase, and Google Scholar reveals several limitations. These include the absence of citation data, heterogeneity in the data, the presence of numerous duplicate records, and dynamic changes in retrieval results. In contrast, WoSCC possesses comprehensive citation relationship data and exhibits significantly superior integrity in the citation network compared to other databases. Concurrently, WoSCC has standardized data processing functions and ensured the compatibility of analysis tools. These tools automatically combine different spelling variants of the same authors and institutions into the same category, thereby reducing the error of data analysis. Additionally, they can directly export the plain text indicator data that supports bibliometric tools. In this study, the literature was screened according to the PRISMA 2020 new systematic evaluation process, and the literature search strategy was as follows: The search string employed was as follows: “TS=(((“stomach”OR”gastric”)AND(“cancer”OR”tumor”OR”oncology”OR”neoplasm”OR”carcinoma”))AND(“peritoneal metastasis”OR”peritoneal carcinomatosis”OR”metastatic peritoneal cancer”OR”peritoneal dissemination”OR”peritoneal seeding”))”. The search period spanned from January 1, 2004, to October 31, 2024. The literature types that were selected for inclusion in this study were limited to articles and review articles. Excluded from the study were meeting abstracts, editorials, proceeding papers, letters, early access publications, corrections, retracted publications, notes, retractions, publications with expressions of concern, and other literature types. The language of the literature has been designated as English. In order to prevent data bias caused by database updates, the search date was set to November 5, 2024, and all literature retrieval, screening, and data extraction were completed within 1 day. The screened literature data were exported in “plain text” format. Given the substantial volume of literature in the database, the collected data were subjected to a de-duplication process utilizing the Citespace version 6.1.R3 function to eliminate duplicates. This was followed by a manual verification of author and institution names by two researchers to ensure the accuracy of the data. Following a thorough screening process, a total of 2,416 relevant publications were identified for inclusion in the analysis. **Figure 1** is a detailed flowchart of the search strategy.

### 2.2 Data analysis

The researcher extracted data from the documents retrieved from the WoSCC database, including country/region, institution, author information, journal, title, abstract, keywords, references, citations, and year of publication, and conducted preliminary screening. The data were analyzed using Citespace version 6.1.R3, developed by Chen Chaomei, and VOSviewer 1.6.20, developed by Nees Jan van Eck and Ludo Waltman. Additionally, the Studio version 4.4.2 and Bibliometrix packages were utilized (14, 15).

Citespace version 6.1.R3 is a Java-based tool that facilitates the visual analysis of scientific literature. The primary function of the system is to transform literary data into comprehensible visual representations through the application of citation analysis, co-occurrence analysis, and time series modeling. Citespace version 6.1.R3 was used to calculate co-citation and citation frequencies, centrality, and keyword bursts and to produce network diagrams of institutions, authors, keyword timeline diagrams, and keyword ranking diagrams. VOSviewer 1.6.20, a bibliometric analysis software, assists researchers in visualizing and comprehending the structure, prominent areas of focus, and prevailing trends within academic domains by establishing a network of associations among documents, authors, institutions, or keywords. The visual analysis was performed by VOSviewer 1.6.20 to map the network of inter-country cooperation, calculate the frequency and centrality of keywords, and plot keyword clusters. The Bibliometrix package, an instrumental tool for bibliometric research, boasts automated data processing capabilities and seamless compatibility with other R language packages. RStudio (version 4.4.2) and the Bibliometrix package were used to analyze and produce maps of national author-co-authored releases, journal releases, and keyword heat maps.

## 3 Results

### 3.1 Analysis of the number of publications and citations

**Figure 2:**
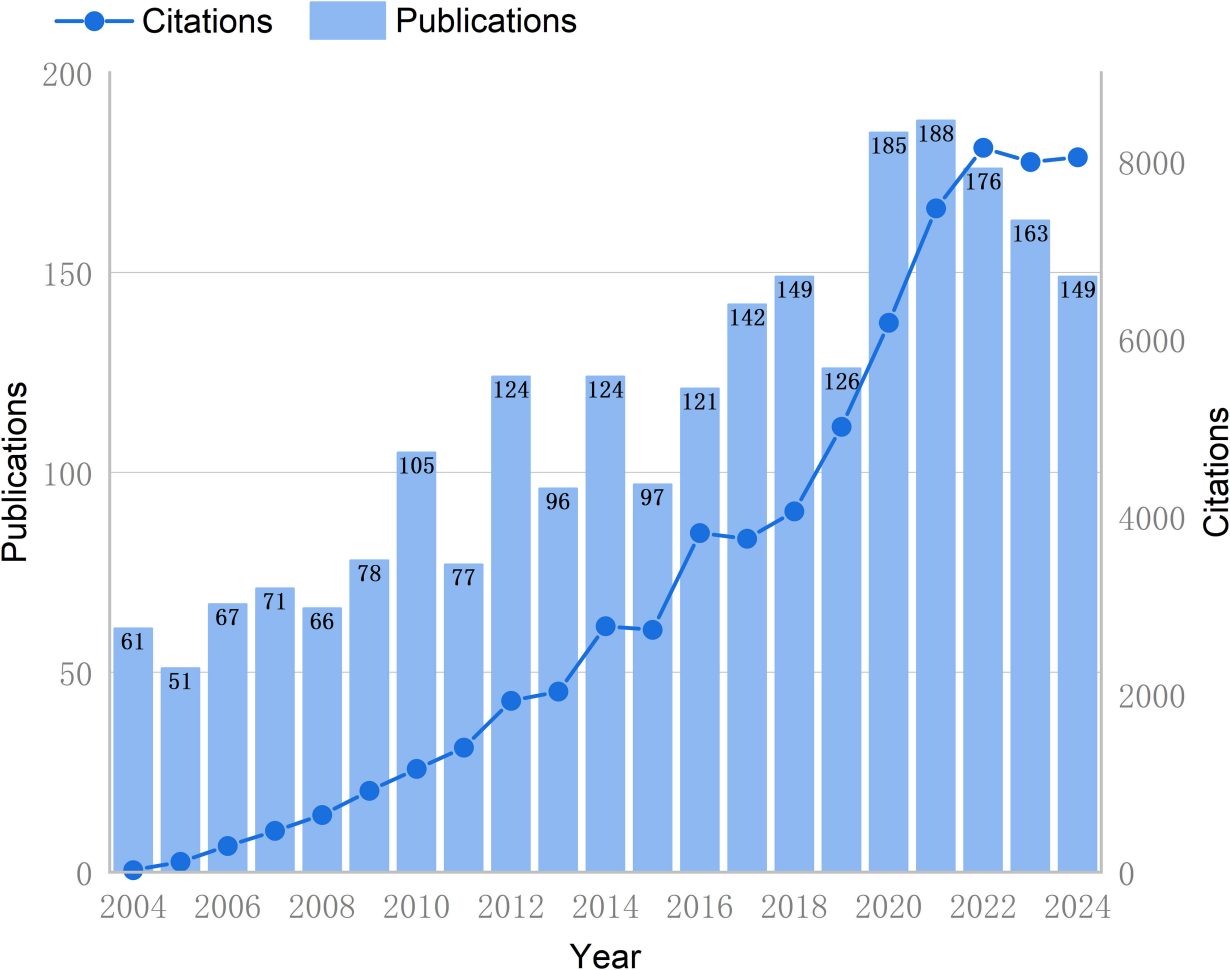
Trend chart of annual publications and literature citations in the GCPM field from 2004 to 2024. The bar graph represents the annual number of publications (left Y-axis), and the line graph shows the cumulative number of citations (right Y-axis).

The researchers’ comprehensive search and screening process yielded 2,416 articles that met the predetermined criteria, which were retrieved from the WoSCC database. As demonstrated in **Figure 2**, since 2004, the number of publications and citations of literature related to GCPM has continued to increase, and then from 2020 to 2024, the growth stabilized. The growth rate of GCPM publications fluctuates between 2004 and 2021, but is generally positive. Results indicate that the years 2012 and 2020 experienced accelerated growth in publications, with respective increases of 61.04% and 46.83%. However, subsequent years following 2021 are forecast to demonstrate a four-year sequence of negative growth.

### 3.2 Analysis of national and international cooperation work

**Figure 3:**
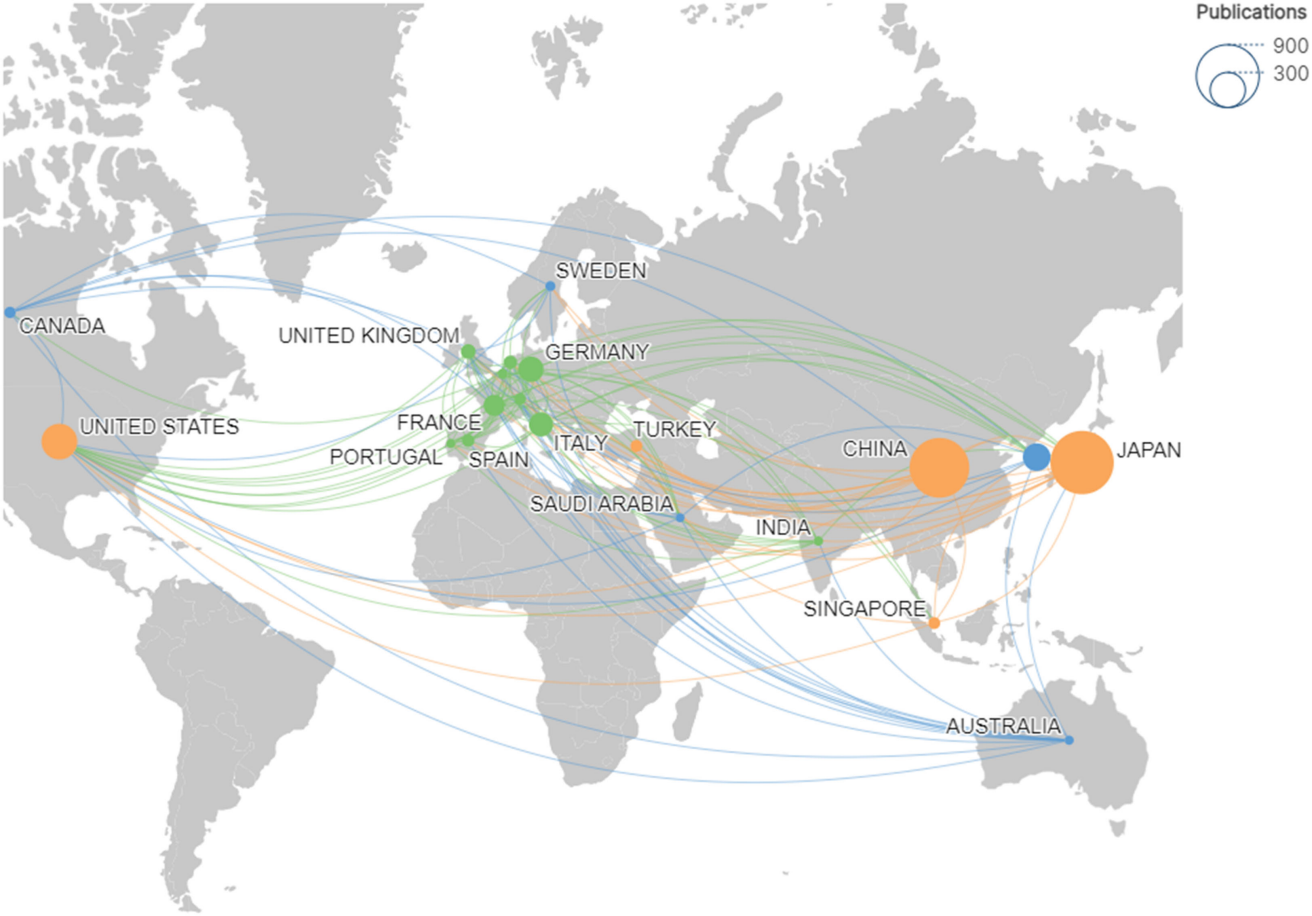
Geographic map of the top 20 countries with publications in the GCPM field. Node size corresponds to publication volume; colors and connecting lines indicate national cooperation networks.

**Figure 4:**
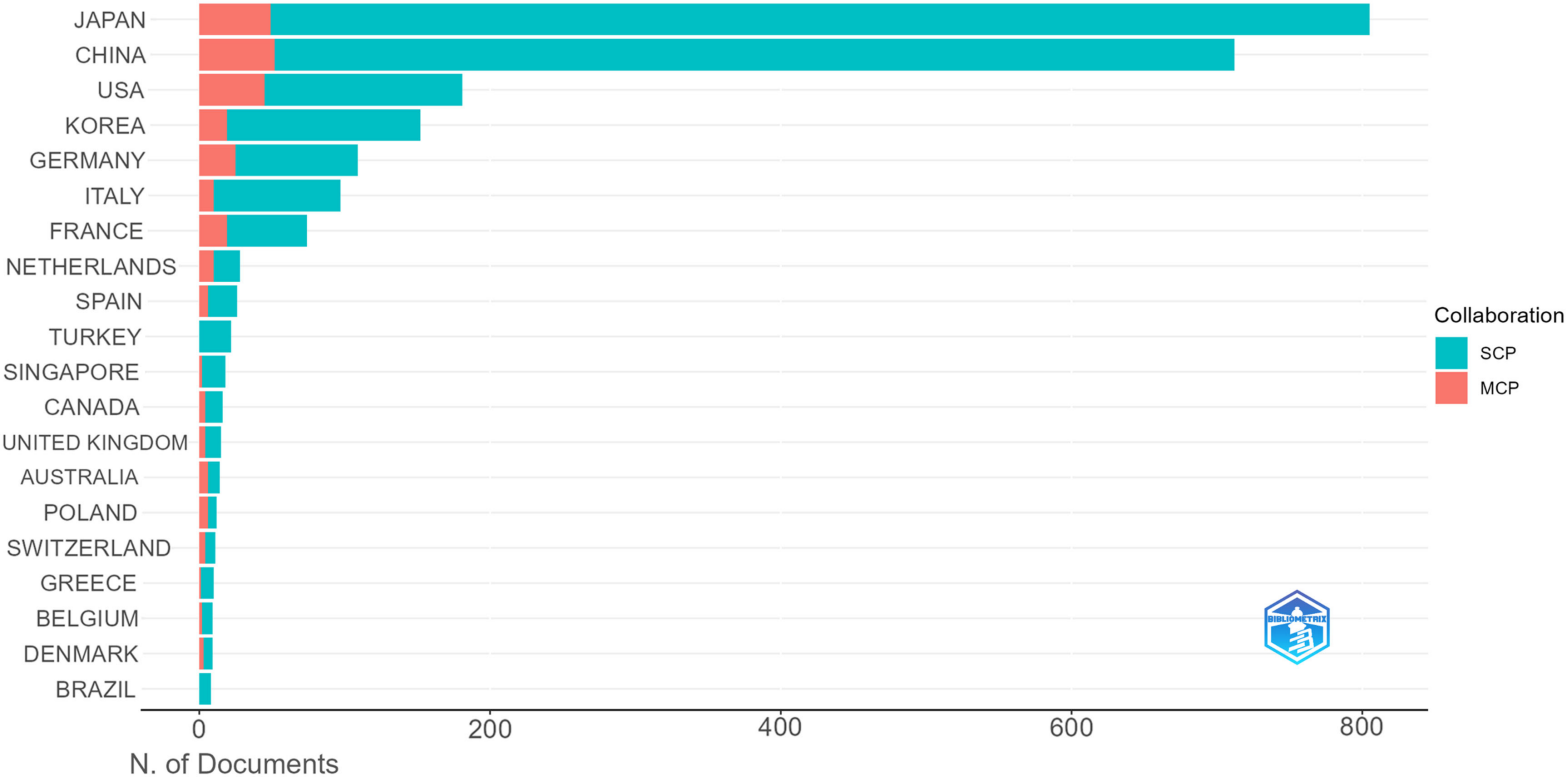
Top 20 corresponding authors’ countries. SCP (Single Country Publication, a publication in which all authors are from the same country); MCP (Multiple Country Publications, which refers to publications co-authored by authors from two or more countries).

**Figure 5:**
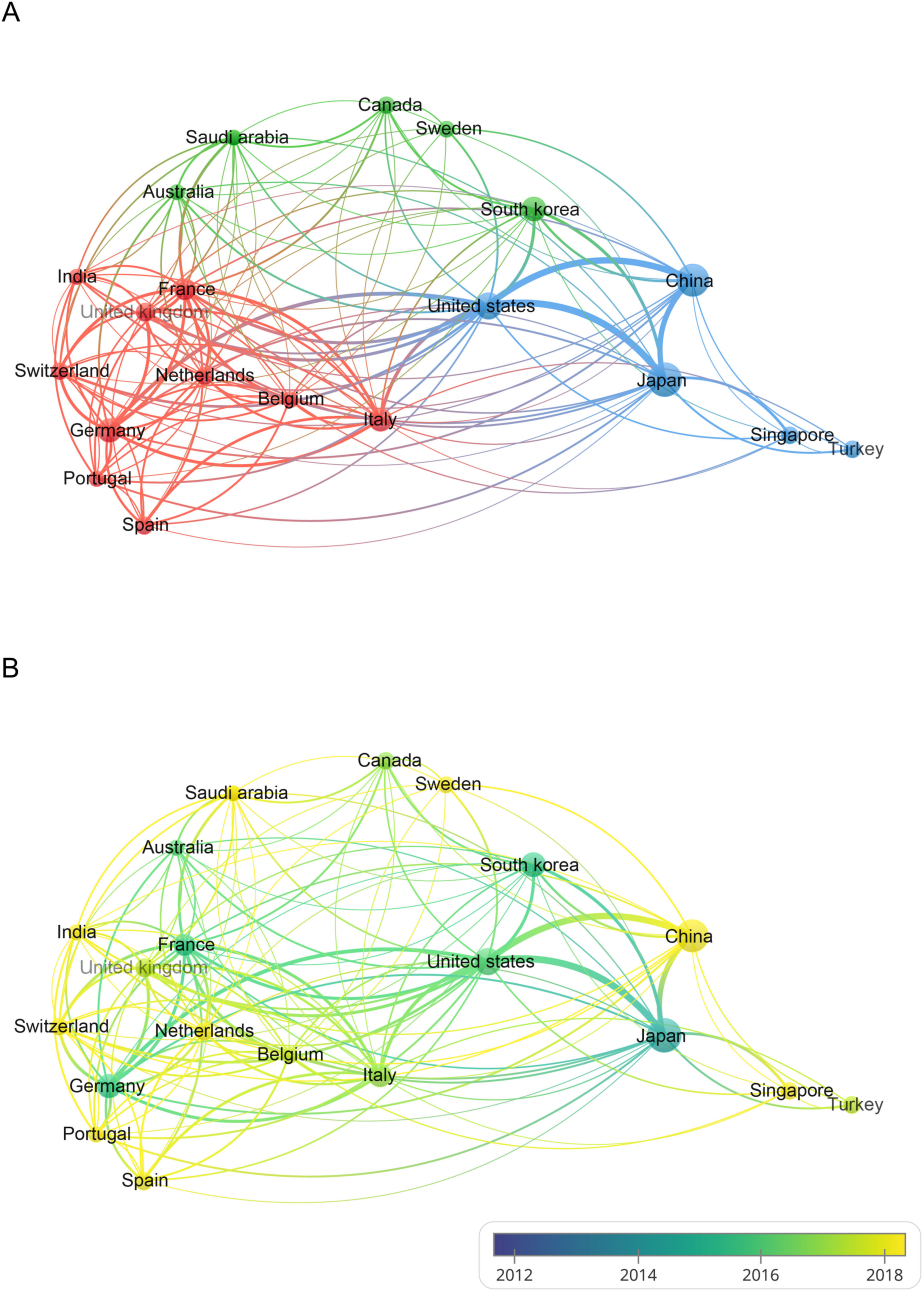
**(A)** Visualization of the cooperation network between the top 20 countries with publications in the field of GCPM. **(B)** Overlay visualization maps based on time changes. The node size indicates the number of publications, the color indicates the cluster to which the country belongs, and the thickness of the line indicates the level of cooperation between countries.

Using Citespace and Scimago Graphica graphical software, geographic co-occurrence maps of synergistic relationships were created by combining keywords belonging to the same country according to the country of publication of the literature. **Figure 3** illustrates the geographical distribution of global publications in the GCPM field by the primary 20 countries and regions. **Table 1** shows that research on GCPM is most common in Japan, with 842 articles published, followed by China with 748 articles, the United States with 268 articles, and South Korea with 161 articles. In bibliometrics, centrality measures the importance of network nodes (e.g., countries, authors, keywords) in an academic network. Country centrality shows that Japan and the United States exhibit comparable academic dominance (0.11). While Italy and the United Kingdom demonstrate higher centrality, they exhibit lower publication volumes. **Figure 4** illustrates the number of papers published by authors from the same country in the top 20 countries in the field of GCPM, in collaboration with authors from multiple countries. Japan, China, and the United States have all demonstrated a propensity for relatively close research collaborations with other countries. **Figure 5** presents an analysis of the co-authors in these 20 countries, utilizing VOSviewer. As demonstrated in **Figure 5B**, China has recently engaged in a substantial number of research collaborations.

**Table 1:** Top 10 countries with the highest productivity.

Rank	Countries	Publications	Centrality	Area
1 2 3 4 5 6 7 8 9 10	JAPAN CHINA USA SOUTH KOREA GERMANY ITALY FRANCE UK NETHERLANDS SPAIN	842 748 268 161 136 124 94 46 38 33	0.11 0.06 0.11 0 0.03 0.15 0 0.21 0.06 0.02	Asia Asia North America Asia Europe Europe Europe Europe Europe Europe

### 3.3 Analysis of the contributions by institutions

**Figure 6:**
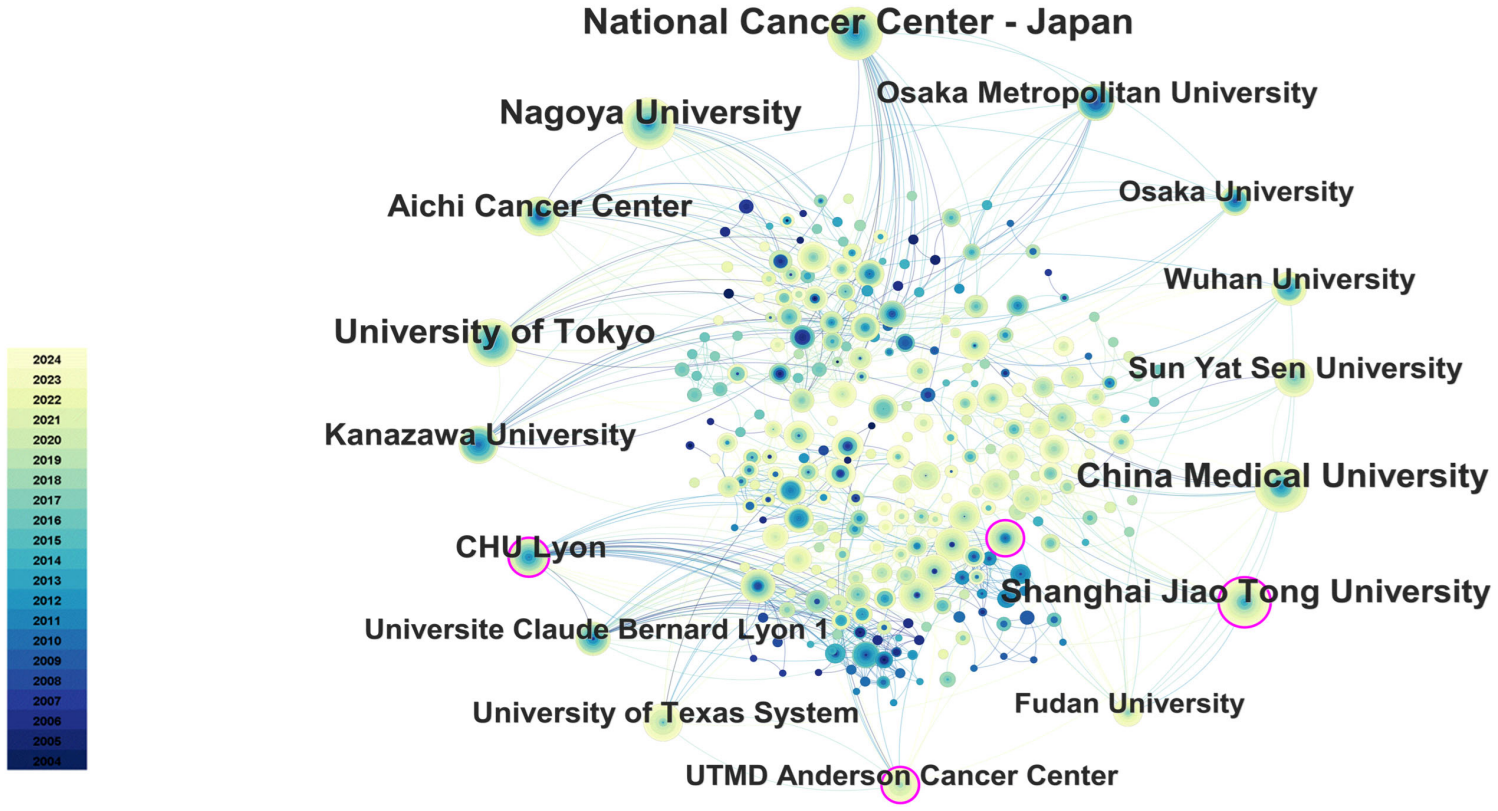
The main collaboration network of institutions. The size of the nodes represents the number of publications, and the color gradient indicates the year of publication (from dark for 2004 to light for 2024). From: CiteSpace.

Citespace software performed a co-occurrence analysis of publishing institutions. A total of 415 institutions have published research in this field. As illustrated in **Table 2**, the leading institutions in terms of productivity are predominantly in Japan and China. The institution that published the most papers was the National Cancer Center-Japan (106 articles), followed by Nagoya University (81 articles) and China Medical University (76 articles). Furthermore, the institution with the highest centrality is CHU Lyon (0.11), followed by UTMD Anderson Cancer Center (0.10), Kanazawa University (0.09), and Shanghai Jiao Tong University (0.08), as illustrated in **Figure 6**.

**Table 2:** The top 10 institutions with high publications.

Rank	Institutions	Publications	Centrality	Country
1	National Cancer Center - Japan	106	0.08	Japan
2	Nagoya University	81	0.01	Japan
3	China Medical University	76	0.07	China
4	University of Tokyo	75	0.04	Japan
5	Shanghai Jiao Tong University	64	0.08	China
6	Aichi Cancer Center	52	0.05	Japan
7	Kanazawa University	48	0.09	Japan
8	CHU Lyon	44	0.11	France
9	Sun Yat-Sen University	43	0.01	China
10	Osaka Metropolitan University	42	0.06	Japan

### 3.4 Analysis of the contributing authors

**Figure 7:**
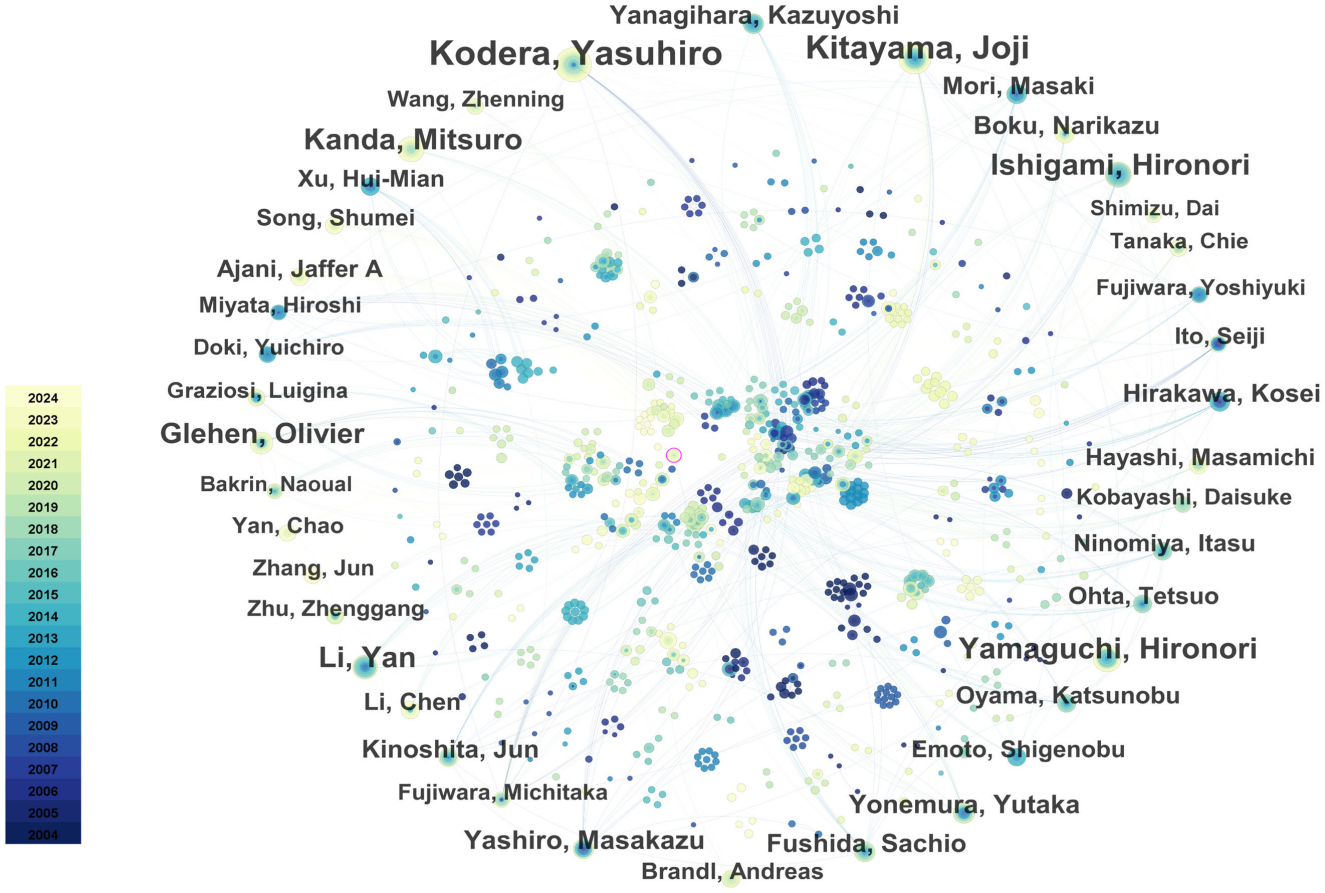
The collaboration network of core authors. The size of the nodes represents the number of publications, and the color gradient indicates the year of publication (from dark for 2004 to light for 2024). From: CiteSpace.

**Figure 8:**
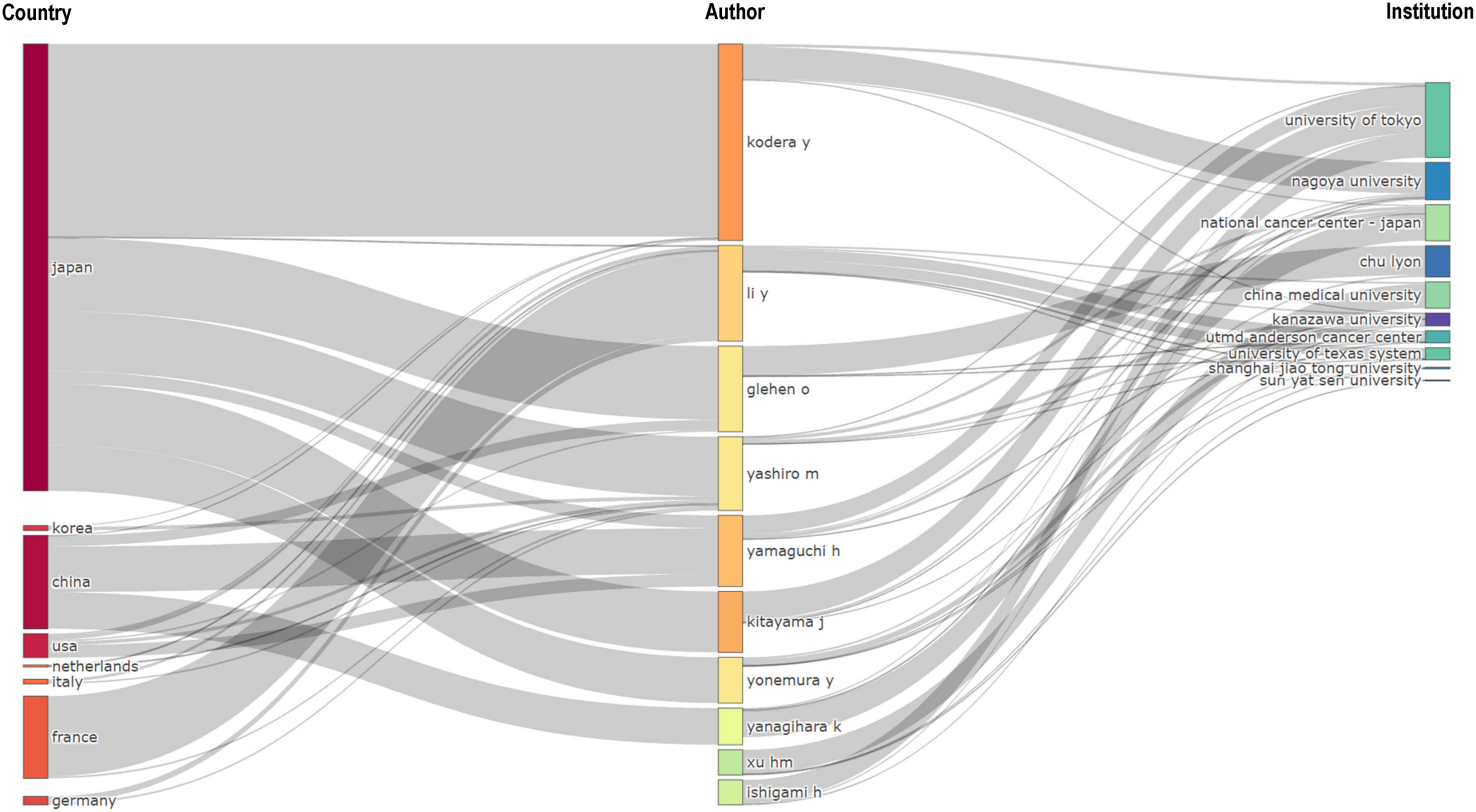
Three-field plot (middle field: authors; left field: countries; right field: institutions). The thickness of the links is positively correlated with the number of scholarly outputs.

Through the co-occurrence analysis of paper authors using Citespace software, 813 researchers have published papers in this field, of which 42 have published 10 or more. Yasuhiro Kodera, Joji Kitayama, Hironori Yamaguchi, Hironori Ishigami, and Mitsuro Kanda are the most prolific researchers. In terms of author centrality, Hideo Baba, Mitsuro Kanda, and Yutaka Yonemura rank highest. Most prolific and influential researchers are from Japan and China. **Table 3** summarizes the top 10 researchers in terms of publication volume and centrality. The co-author network diagram is shown in **Figure 7**. **Figure 8** shows the interconnections between research countries, institutions, and authors.

**Table 3:** The top 10 most productive authors.

Rank	Authors	Publications	Centrality	Year
1	Kodera, Yasuhiro	60	0.03	2007
2	Kitayama, Joji	47	0.05	2009
3	Yamaguchi, Hironori	32	0.01	2011
4	Ishigami, Hironori	31	0	2009
5	Kanda, Mitsuro	30	0.09	2016
6	Li, Yan	29	0.01	2008
7	Glehen, Olivier	27	0.06	2010
8	Yashiro, Masakazu	20	0.05	2006
9	Yonemura, Yutaka	19	0.08	2009
10	Yanagihara, Kazuyoshi	19	0.01	2008

### 3.5 Analysis of the contributions of journals

**Figure 9:**
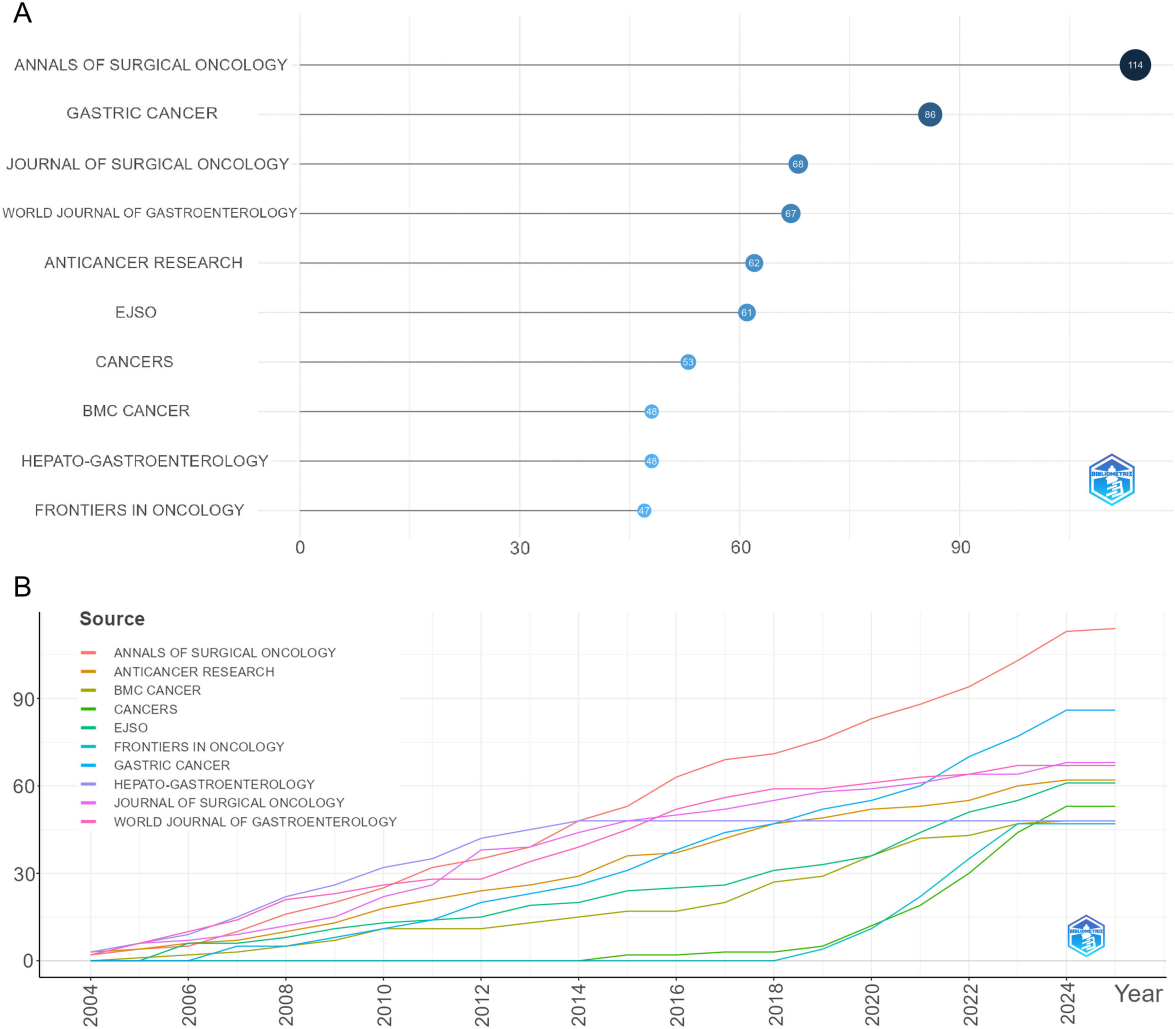
Visualization of journal analysis. **(A)** The top 10 journals by publication volume. **(B)** The growth trend of journal publications.

**Figure 10:**
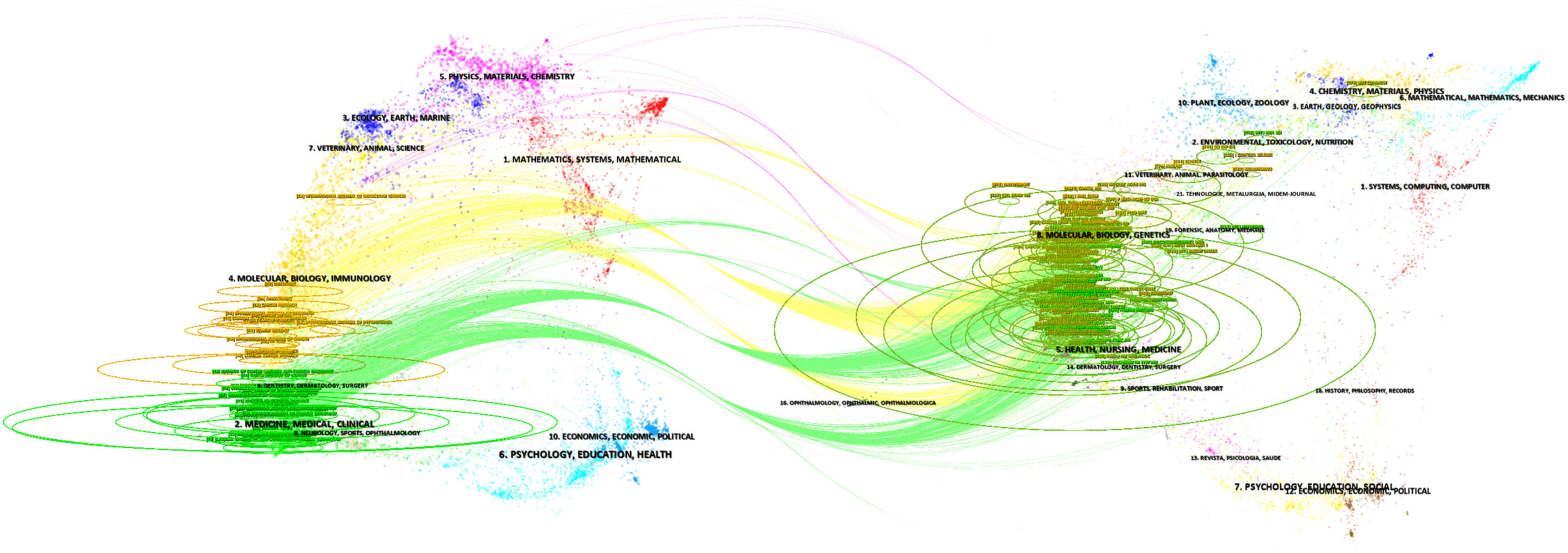
The dual-map overlay of journals related to GCPM. The left area represents citing journals, and the right area represents cited journals; the color of the lines indicates different disciplines.

A total of 200 journals have published research related to GCPM. As illustrated in **Figure 9**, the figure displays the total and annual publications of the ten most prolific journals in the field. **Table 4** illustrates the top 10 cited journals. The researchers employed Citespace to generate a double-map overlay atlas, illustrating the distribution of citing and cited journals. As illustrated in **Figure 10**, the left area corresponds to the citing journal, while the right area represents the cited journal. The color of the lines serves as a symbol of the different disciplines. The journals above predominantly encompass the domains of molecular biology, genetics, and nursing, while others primarily address clinical medicine, surgery, molecular biology, and immunology.

**Table 4:** The top 10 most cited journals.

Rank	Full journal title	Citations	IF2023	WOS Categories
1	Annals of Surgical Oncology	1270	3.4	SURGERY
2	Journal of Clinical Oncology	1261	42.1	ONCOLOGY
3	Gastric Cancer	1142	6	GASTROENTEROLOGY & HEPATOLOGY
4	Journal of Surgical Oncology	1047	2	SURGERY
5	Annals of Surgery	945	7.5	SURGERY
6	Cancer Research	933	12.5	ONCOLOGY
7	British Journal of Surgery	925	8.6	SURGERY
8	International Journal of Cancer	892	5.7	ONCOLOGY
9	Cancer-AM Cancer SOC	851	6.1	ONCOLOGY
10	World Journal of Gastroenterology	788	4.3	GASTROENTEROLOGY & HEPATOLOGY

### 3.6 Analysis of a highly cited study

**Figure 11:**
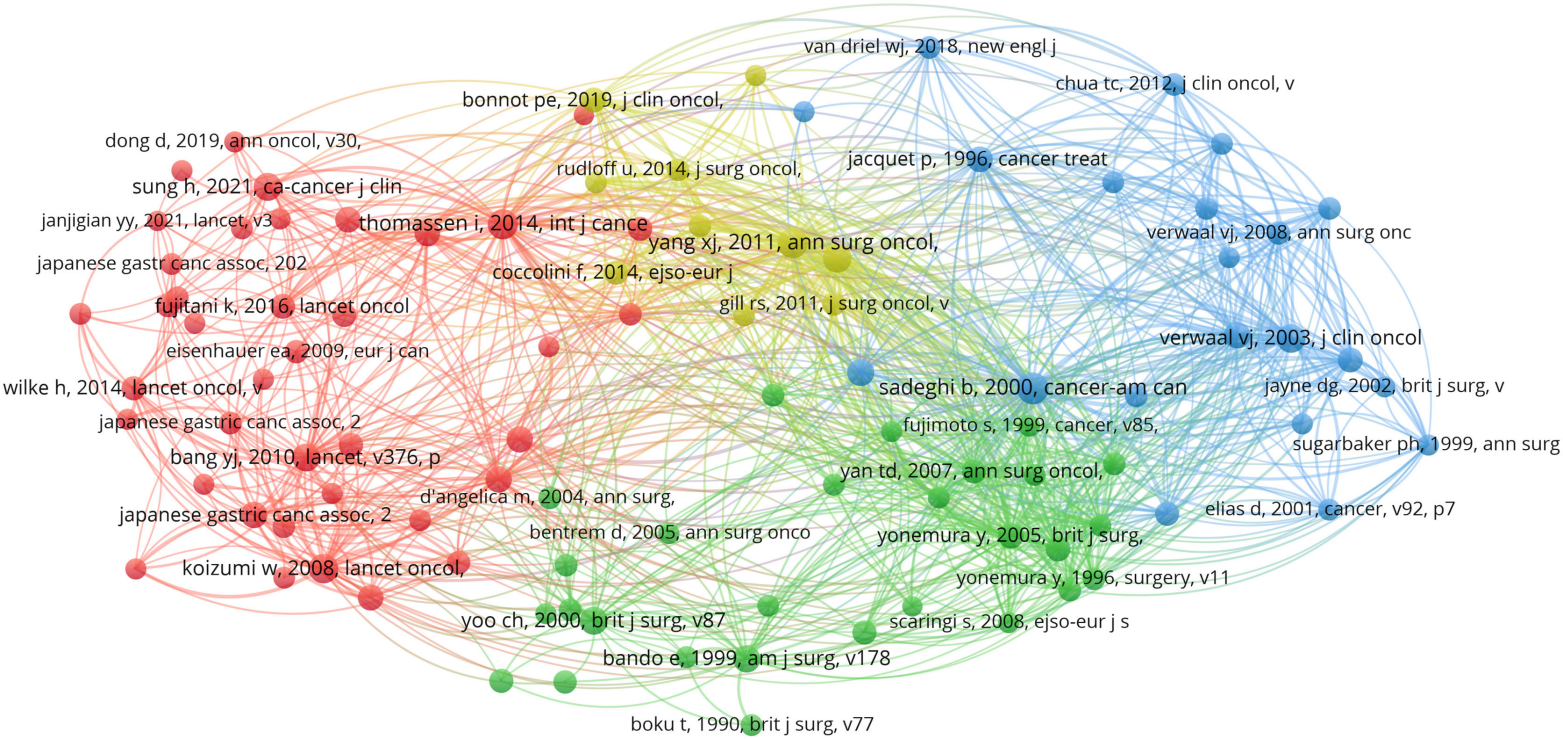
Visualization map of co-cited references. Node size indicates the frequency of occurrence of the cited literature, and lines between nodes indicate the presence of co-occurrence.

**Table 5** presents a list of the five articles that have been cited most frequently. The article entitled “Japanese Gastric Cancer Treatment Guidelines 2014 (ver. 4)” has been identified as the most frequently cited and published in the journal Gastric Cancer. A comprehensive analysis of the pertinent references was conducted, resulting in the selection of articles that had garnered more than 50 citations, as illustrated in **Figure 11**.

**Table 5:** The top 5 co-citation references related to GCPM.

Rank	Title	First author	Source	Citations
1	Japanese gastric cancer treatment guidelines 2014 (ver. 4)	Japanese Gastric Cancer Association	Gastric Cancer	2076
2	Current treatment and recent progress in gastric cancer	Smita S Joshi	CA: A Cancer Journal for Clinicians	988
3	Gastrectomy plus chemotherapy versus chemotherapy alone for advanced gastric cancer with a single non-curable factor (REGATTA): a phase 3, randomized controlled trial	Kazumasa Fujitani	The Lancet Oncology	526
4	Gastric cancer: ESMO Clinical Practice Guideline for diagnosis, treatment and follow-up	F Lordick	Annals of Oncology	524
5	Cytoreductive surgery and hyperthermic intraperitoneal chemotherapy improves survival of patients with peritoneal carcinomatosis from gastric cancer: final results of a phase III randomized clinical trial	Xiao-Jun Yang	Annals of Surgical Oncology	505

### 3.7 Analysis of keywords

**Figure 12:**
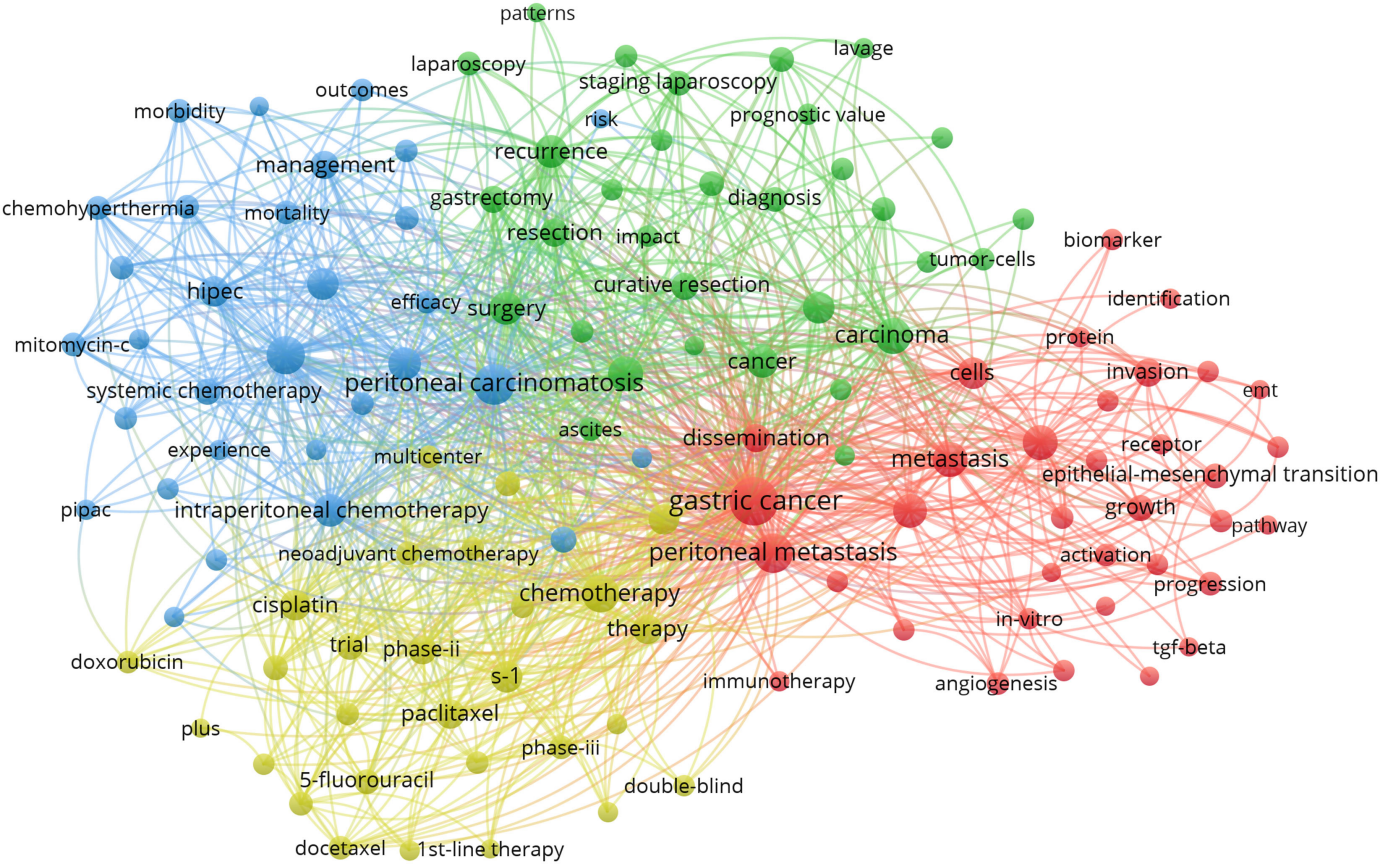
Keyword co-occurrence networks in the GCPM field. Node size indicates the frequency of keyword occurrence; lines between nodes indicate the presence of co-occurrence relationships; and the keyword co-occurrence network is divided into four color-coded clusters based on the evolution of research hotspots.

**Figure 13:**
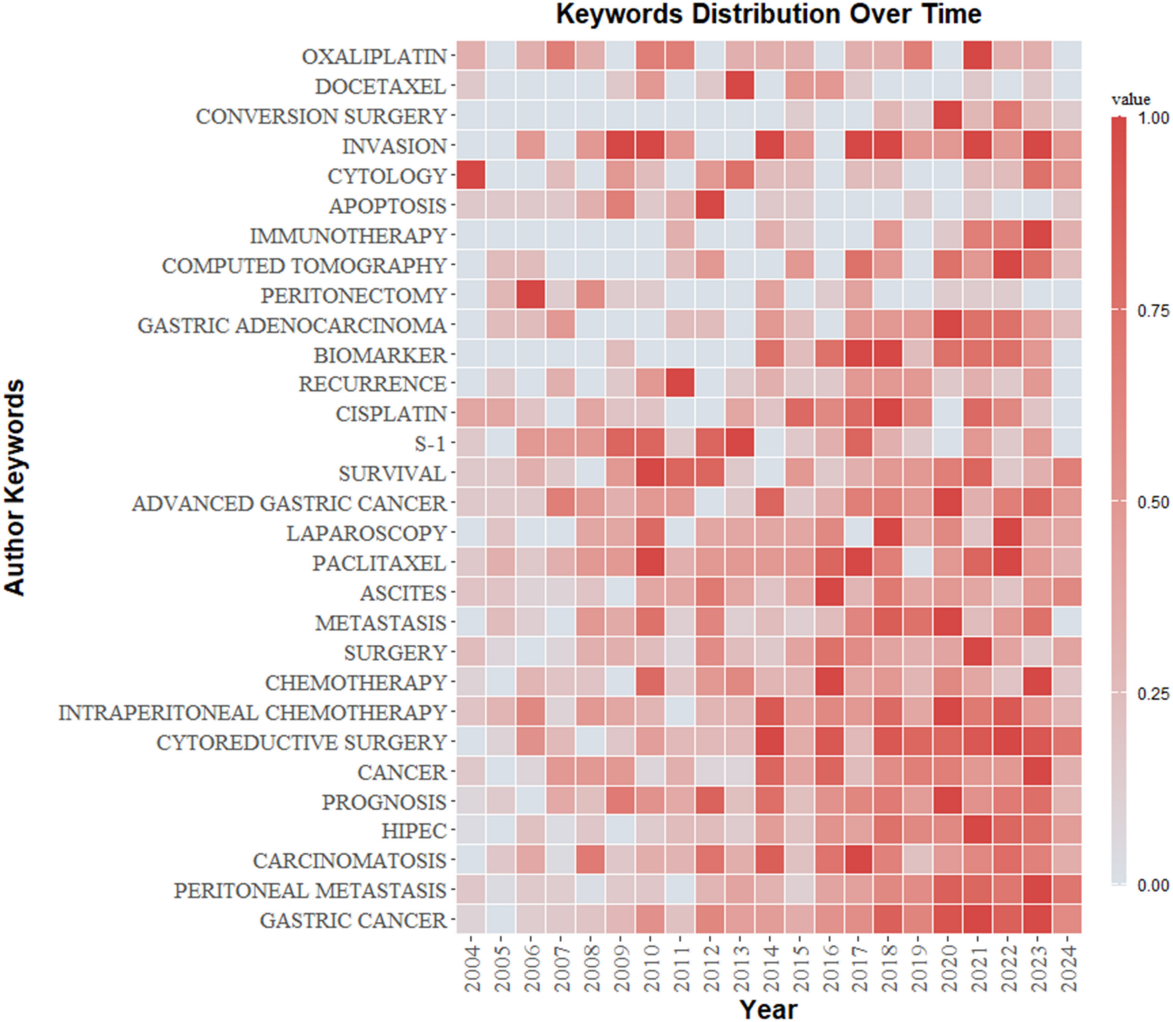
Keywords heat map. The graph shows how the frequency of keyword occurrences varies according to time. The color gradient goes from blue (low frequency) to red (high frequency).

**Figure 14:**
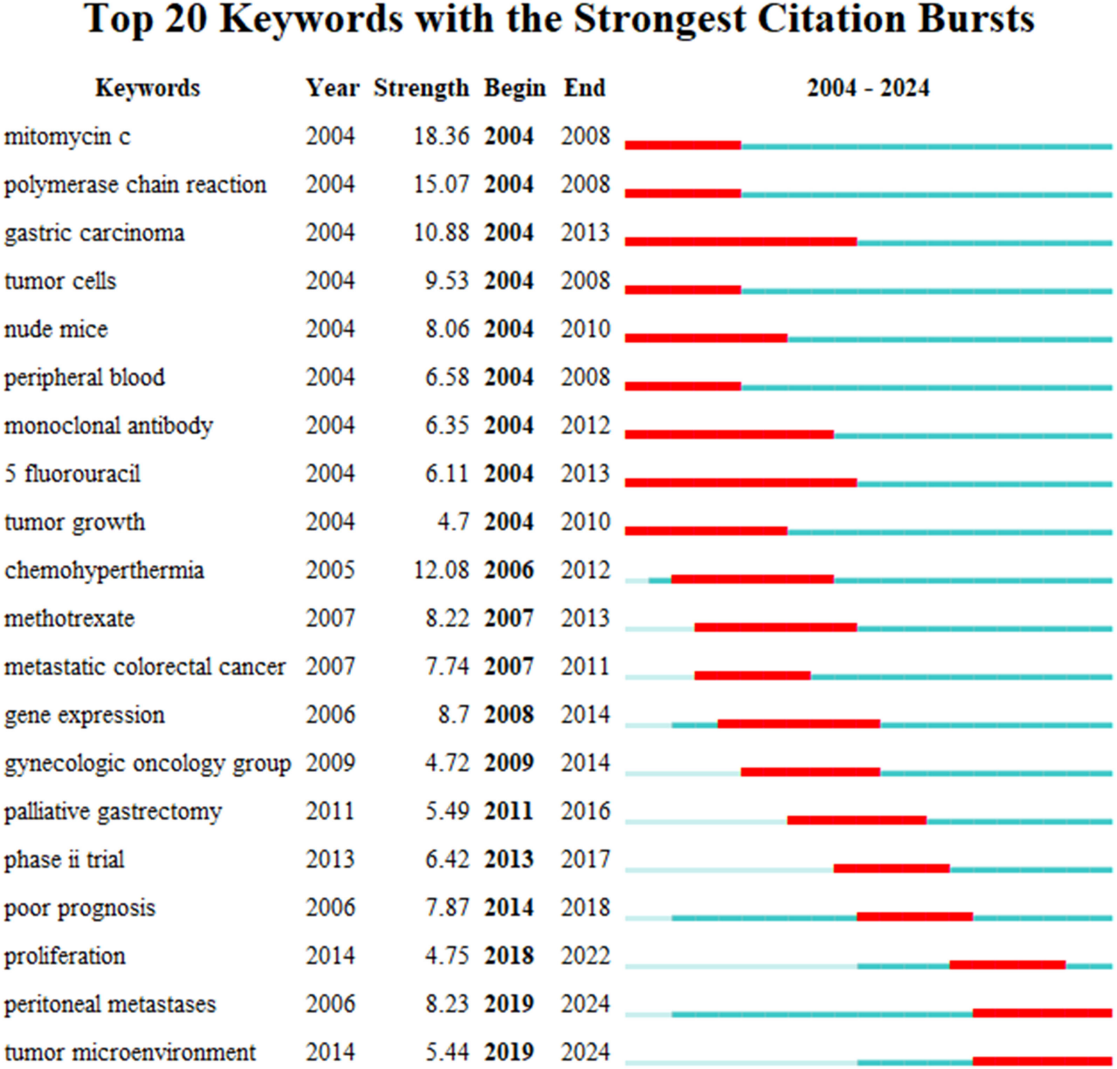
Top 20 keywords with the strongest citation bursts. The bar chart highlights keywords that have experienced significant surges in citations over specific time .s. The length and color of the bars indicate the duration and intensity of the citation bursts, respectively.

**Figure 15:**
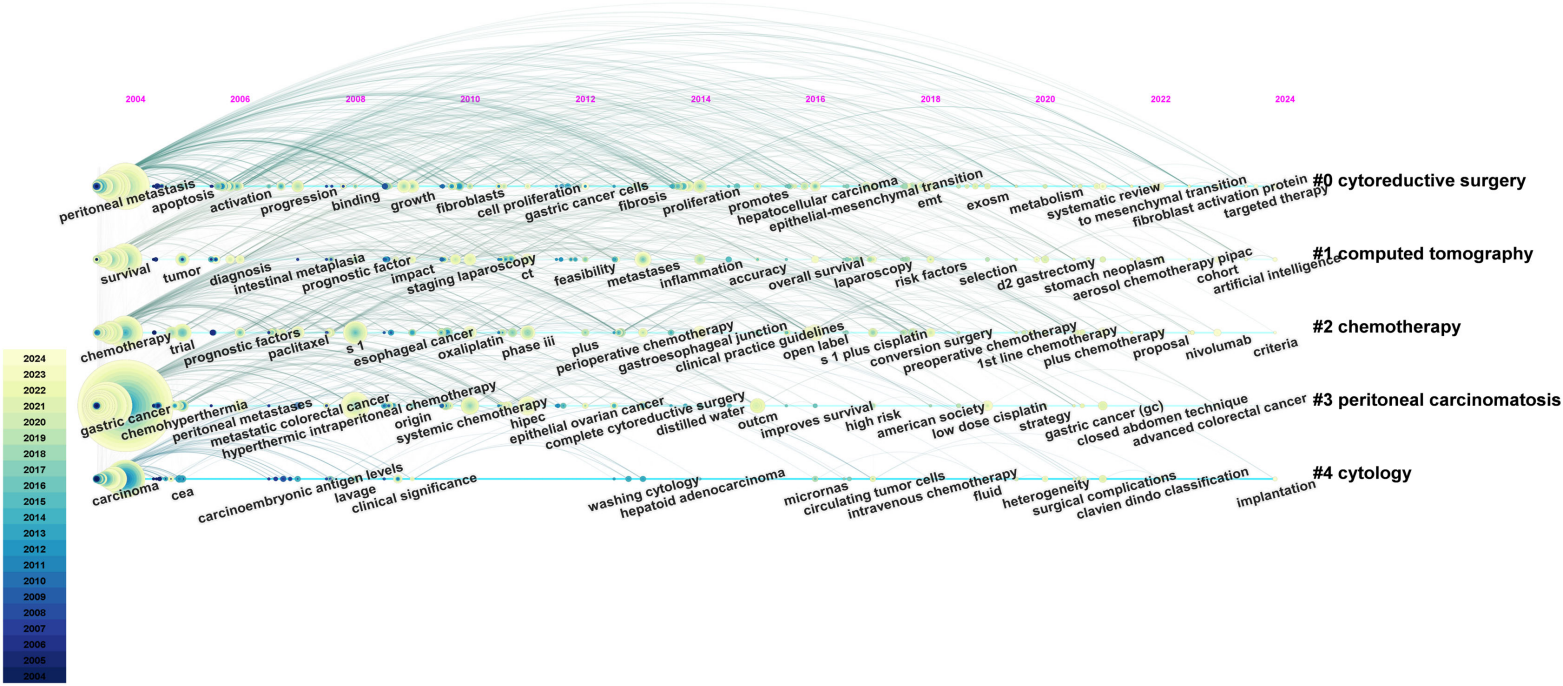
A timeline and keyword clustering display for the GCPM. This timeline graphically illustrates the evolution of research hotspots in the field of GCPM, with cluster sizes indicating the frequency of keyword occurrences and connecting lines indicating changes in research trends over time. From: CiteSpace.

VOSviewer facilitated the construction of the keyword network graph. From a total of 6,422 keywords, we selected 127 high-frequency keywords exhibiting a frequency of occurrence greater than 30 times. As illustrated in **Figure 12**, the keyword clustering network categorizes the screened keywords into four distinct clusters. Cluster #1 (red area, RGB code: #D62728): related mechanisms of GCPM, mainly including gene, protein, biomarker expression, vascular invasion, and tumor angiogenesis; Cluster #2 (green area, RGB code: #2CA02C): prognosis of GCPM, including recurrence, prognostic factors, surgical resection, and diagnostic methods; Cluster #3 (yellow area, RGB code: #BCBD22): chemotherapy of GCPM, including platinum, paclitaxel, S-1, and neoadjuvant chemotherapy; Cluster #4 (blue area, RGB code: #1F77B4): intraperitoneal treatment of GCPM, including intraperitoneal perfusion chemotherapy, intraperitoneal hyperthermic perfusion chemotherapy, and cytoreductive surgery. The researcher used R language to analyze the 30 keywords with the highest frequency of occurrence and create a keyword heat map, as illustrated in **Figure 13**. **Figure 14** illustrates the top 20 most frequently cited keywords in this field. The analysis of the outbreak intensity of keywords reveals significant fluctuations between 2004 and 2013, with higher intensity observed for “mitomycin c” and “polymerase chain reaction” in the early stage and “chemo hyperthermia” in the middle stage. Thereafter, there is a decline in overall intensity. In the early period, “mitomycin C” and “polymerase chain reaction” exhibited higher intensity, while “chemo hyperthermia” dominated in the middle period, and the overall intensity value decreased after 2013. **Figure 15** presents a visual representation of the timeline of keywords in GCPM from 2004 to 2024, thereby illustrating the dynamic evolution of these terms. In the early stage, research focused on the exploration of treatment modalities such as “chemotherapy” and “HIPEC” (hyperthermic intraperitoneal chemotherapy). In the middle stage, research shifted to the study of molecular mechanisms such as “EMT” (epithelial-to-mesenchymal transition). In the mid-term, the research focuses on molecular mechanisms such as EMT, while in the recent period, the hotspots focus on “artificial intelligence” and “targeted therapy.”

## 4 Discussion

### 4.1 Global research status and trends

A statistical analysis of the number of national publications shows that Japan, China, and the United States have the largest number of publications, accounting for 78.3% of publications in the field of GCPM. Japan has emerged as a dominant contributor, with 842 articles and a high level of centrality. The National Cancer Center-Japan and other institutions have established a close cooperation network, fostering collaborative research and knowledge exchange. Despite abundant literature on China, its centrality remains comparatively low, indicating a potential deficit in the depth of international cooperation. European countries such as Italy and the United Kingdom have a limited number of articles but high centrality and outstanding academic influence. The top five authors, all from Japan, have demonstrated a high degree of collaboration, as evidenced by the substantial number of their publications and the author network diagram, which suggests the formation of a close and influential group of authors in Japan. From the perspective of journal publication volume and citation volume, Annals of Surgical Oncology has the largest number of publications and citations, making it an important journal in the field of GCPM research, and its papers have important reference value. An analysis of articles related to GCPM research indicated that the Japanese gastric cancer treatment guidelines 2014 (ver. 4), published by the Japanese Gastric Cancer Association, was the most cited article in this field. This literature is a clinical practice guideline that provides standardized treatment options for metastatic GC by standardizing the peritoneal metastasis staging system and integrating surgery, chemotherapy, and targeted therapies, making it an important reference for international GC treatment with a profound impact on clinical practice, especially in Asian populations. However, this guideline version did not include immunotherapy, and subsequent studies need to be supplemented by following the latest version or reviewing the literature on immunotherapy in GCPM.

Different epidemiologic profiles and strategic focuses of cancer research are largely to blame for the East-West research gap. GC manifests at a notably high rate within East Asian populations, thus prompting extensive research on GC in Japan and China. Japan’s research hotspots concentrate on the molecular mechanisms, prognostic factors, and development of new treatments for GCPM. The multicenter clinical trials conducted by the institution are of a high caliber, and the research results have a significant international impact, particularly in the domain of technical innovation in the treatment of GC. China’s research initiatives encompass a range of areas, including epidemiology, early diagnosis, the development of prognostic models, and comprehensive treatment strategies for GCPM. China has a substantial number of GC patients, which facilitates large-scale cohort studies and bioinformatics analysis, underscoring its immense potential in clinical research and translational medicine. The United States has a relatively small number of GC patients, and although it is a leader in targeted and immunotherapy innovations, the efficiency of clinical translation is limited by the number of cases. Concurrently, disparities in standardized treatment protocols between Eastern and Western regions have led to constrained clinical trial generalizability. Changes in publication growth rates also reflect the stage-by-stage characteristics of GCPM research. Early rapid growth stemmed from mechanism breakthroughs and therapeutic innovations. The recent slowdown suggests the need to promote a new round of breakthroughs through interdisciplinary integration and international cooperation. The advent of innovative diagnostic and therapeutic methodologies, such as AI and multi-omics technology, necessitates a standardized validation system. In the future, it is necessary to build a global collaborative network, integrate the strengths of various countries, and promote trans-regional multi-center trials to break through the current therapeutic bottlenecks.

### 4.2 Research keyword analysis

Keyword clustering analysis indicates that the nodes of emerging therapeutic modalities such as “PIPAC” and “immunotherapy” are isolated and sparse, suggesting that the research has not yet formed a system that can be a focus of clinical translation in the future. Fewer nodes in diagnostic technology clusters indicate a paucity of innovative diagnostic modalities, which merits further investigation. A thorough examination of the keyword outbreak intensity graph and timeline graph reveals a gradual shift in the research focus and hotspot of GCPM from early basic treatment to emerging diagnostic and therapeutic modalities. However, traditional therapeutic means remain the core. The keyword outbreak intensity of the basic research-related keywords declined after 2013, and the high-frequency keywords continued to prioritize the diagnosis of peritoneal metastasis and comprehensive treatment, which suggests the necessity for future efforts to enhance the clinical translation and integration of emerging technologies.

#### 4.2.1 Cluster#1: molecular mechanisms of GCPM

GCPM follows the “seed-soil theory”: the “seed” is detached GC cells, and the “soil” is the peritoneal microenvironment. Plantation metastasis is the main pathway of GCPM. Cancer cells infiltrate and penetrate the plasma membrane layer of the gastric wall to the peritoneal cavity or directly invade the adjacent peritoneal tissues. Recent studies have found that mutations in ELF3, CDH1, PIGR, and other genes are closely related to peritoneal metastasis from primary lesions. Mutations in E-cadherin encoded by CDH1, an important molecule that maintains the adhesion of epithelial cells, will decrease the adhesion ability of the cells and induce EMT of the cancer cells, enhancing their invasive ability (16, 17). Studies have shown that the primary GC of the EMT subtype classified according to the Asian Cancer Research Group is associated with diffuse GC, and the risk of peritoneal metastasis and prognosis is significantly worse than those of the non-EMT subtype (18, 19). Multi-omics analysis confirmed the existence of a pre-metastatic niche in the peritoneum of patients with early GC, suggesting that the EMT-related pathway can be used as a predictive marker (17).

Cancer cells from primary foci or metastatic lymph nodes are shed into the peritoneal cavity or widely disseminated with the flow of ascites. Cancer-associated fibroblasts (CAFs) have been shown to promote the formation of metastatic foci in the peritoneum by degrading the extracellular matrix (ECM) through the secretion of matrix metalloproteinases (MMPs). This process occurs when cancer cells disseminate to the peritoneum. CAFs also promote the fibrosis of the ECM through factors such as transforming growth factor-beta (TGF-β), which provides physical support for the cancer cells after EMT (17, 20). Wang X et al. found that the high expression of fibrillin-1 (FBN1) in advanced GC and its succinylation modification prevented its degradation by MMPs and activated the TGF-β1 and intracellular PI3K/Akt pathways to promote peritoneal fibrosis and tumor cell adhesion (21–23). Monoclonal antibodies targeting the FBN1 succinylation site inhibit this process and may serve as potential therapeutic targets.

Peritoneal microenvironment alterations promote peritoneal implantation and immune escape of tumor cells. Factors such as TGF-β and interleukin 10 (IL-10) inhibit immune responses in the peritoneal microenvironment, and GC cells escape immune surveillance through high expression of PD-L1 (17, 24). Vascular endothelial growth factor (VEGF) and others in malignant ascites induce mesothelial cell contraction and peritoneal basement membrane exposure, facilitating tumor cell-peritoneal implantation. Proinflammatory cytokines in malignant ascites can promote the formation of GCPM through the JAK/STAT3 signaling pathway (25). Tumor cells can also secrete signaling molecules in the tumor microenvironment through paracrine and autocrine pathways to promote tumor metastasis (26, 27).

#### 4.2.2 Cluster#2: prognosis of GCPM

The median survival of GCPM patients is only 3–6 months, mainly due to late diagnosis and missed optimal treatment. Early identification and prediction of GCPM is crucial to improving patient prognosis. The low sensitivity (less than 60%) of GC intraoperative peritoneal lavage cytology detection for GCPM limits its clinical application. Consequently, investigators developed an intelligent detection method based on stimulated Raman molecular cytology (SRMC). This method utilizes an AI algorithm to identify signature cell clusters rapidly. Compared to traditional lavage cytology, it exhibits noticeably higher sensitivity and specificity. Its rapid and accurate features are expected to be a key tool for intraoperative staging of GC (28). In the domain of imaging, the integration of CT-based column line drawing and AI models has been demonstrated to enhance the efficacy of early GCPM detection to a substantial degree. The imaging histology column line drawing developed by Dong D and the PMetNet model (AUC values of 0.856 and 0.843, respectively) by Jiang Y’s team has been shown to identify occult peritoneal metastases effectively (29–31). A research team developed a multimodal risk stratification assessment (RSA) model integrating radiomics and clinical data. This model accurately identifies high-risk patients and predicts the risk of peritoneal metastasis and recurrence through the AI fusion of multidimensional data (32).

In terms of prognostic assessment, YuQin Sun et al. constructed gastric cancer peritoneal metastases signatures (GCPMs) through the GSE62254 database and found that the key gene SYNPO2 could be used as a prognostic marker for GCPM. Patients exhibiting high expression of this gene demonstrated unfavorable prognoses and minimal benefit from chemotherapy, while those with low expression exhibited heightened sensitivity to chemotherapy (33). The Peritoneal Cancer Index (PCI) and the Japanese Classification of Gastric Carcinoma (JCGC) are frequently utilized indicators (34). A study indicated that the rate of PCI change based on CT predicted overall survival after chemotherapy in patients with GCPM (35). The PMN model established by Chen QY has been shown to have more clinical advantages than the traditional JCGC staging (36). Furthermore, the integration of hematological indicators with clinicopathological parameters has emerged as a novel approach to survival prediction (37).

#### 4.2.3 Cluster#3: chemotherapy of GCPM

Systemic chemotherapy remains the basis of GCPM treatment. Commonly used chemotherapy drugs include fluorouracil, platinum, and paclitaxel. The first-line treatment for metastatic GC usually adopts XELOX, FOLFOX, and SOX regimens (11). There are differences between the neoadjuvant treatment mode of locally advanced GC between the East and the West. Perioperative chemotherapy is more commonly used in Europe and the United States, while R0D2 radical surgery + postoperative adjuvant therapy is the main mode in Asia. AIO-FLOT3 shows that surgery after neoadjuvant chemotherapy can significantly improve the survival of patients with limited GC metastasis (38). The follow-up clinical trial FLOT4 further showed that compared with the ECF regimen of the MAGIC trial, the FLOT regimen significantly improved patients’ disease-free survival time and overall survival time (39). Targeted therapies guided by genetic testing offer new treatment options for patients with advanced GC. Trastuzumab in combination with chemotherapy is the current first-line treatment option for HER2-positive advanced GC, and ramucirumab alone or in combination with paclitaxel as well as apatinib mesylate are also recommended as second- or third-line treatment options for advanced GC (40–43). In addition, Zolbetuximab, a novel targeted drug against the new GC target Claudin 18.2, more significantly prolonged the survival of patients with Claudin 18.2-positive advanced GC, suggesting that patients with peritoneal metastases may benefit from the treatment (44). In terms of immunotherapy, patients with high PD-L1 expression (combined positive score, CPS ≥ 5) or microsatellite instability-high/deficient mismatch repair (MSI-H/dMMR) showed significant benefit. The CheckMate 649 study indicated that nivolumab in combination with chemotherapy significantly improved overall survival/progression-free survival (OS/PFS) compared to chemotherapy alone in patients with PD-L1 CPS ≥ 5 (45). The KEYNOTE-059 study found that pembrolizumab demonstrated a durable effect on OS/PFS in multilined-treated advanced GC patients, showed durable antitumor activity, and had a manageable safety profile, especially in patients with high PD-L1 expression and MSI-H (46). Current research focuses on combination therapy. Domestic and foreign researchers have conducted a series of clinical trials on immunologic and targeted drugs with combination drugs and have made some progress, but clinical efficacy needs to be further explored (47–49).

#### 4.2.4 Cluster#4: intraperitoneal treatment of GCPM

The plasma-peritoneal barrier limits the efficacy of systemic chemotherapy for GCPM. Meanwhile, GCPM often triggers complications such as malignant ascites, intestinal obstruction, and malignant disease, which makes it difficult for patients to tolerate systemic therapy. Intraperitoneal perfusion chemotherapy can circumvent systemic toxicity by localizing the high concentration of drugs to act directly on the peritoneal lesions. However, the penetration of intraperitoneal chemotherapy is weak, and to enhance the efficacy, measures such as increasing the temperature, pressure, or frequency can be used to optimize drug penetration.

The CYTO-CHIP study demonstrated that cytoreductive surgery (CRS) combined with HIPEC significantly improved OS and recurrence-free survival (RFS) with a comparable safety profile compared to CRS alone (50). A multicenter, phase III clinical trial, GASTRIPEC-I, was designed to evaluate the impact of HIPEC on OS after CRS in patients with GCPM. Although the trial was terminated early due to slow enrollment, follow-up analyses showed more significant improvements in PFS and metastasis-free survival (MFS) in the CRS+HIPEC group without increased risk of adverse events (51).Although HIPEC showed potential in improving PFS, the GASTRIPEC-I trial showed no significant benefit in OS, possibly stemming from patient selection bias. A meta-analysis confirmed that HIPEC improves 3- and 5-year OS and reduces recurrence rates, but its potential risk of renal impairment needs to be cautioned (52). Normothermic intraperitoneal chemotherapy (NIPEC) enables minimally invasive and continuous treatment with implantable chemotherapy pumps and has a milder safety profile than HIPEC (53). Pressurized intraperitoneal aerosol chemotherapy (PIPAC) utilizes aerosol technology to enhance drug penetration, and several studies have confirmed its safety and efficacy in controlling peritoneal tumors (54, 55). Although PIPAC can enhance drug penetration, it faces limitations, such as the uneven distribution of the drug on the peritoneal surface, and only some patients achieve the expected efficacy. Currently, several prospective studies are further evaluating the efficacy of PIPAC, and these studies are expected to provide a solid scientific basis for the efficacy of PIPAC in the treatment of GCPM (56, 57).

Systemic therapy combined with intraperitoneal chemotherapy and neoadjuvant intraperitoneal and systemic chemotherapy (NIPS) has shown favorable therapeutic effects and safety in many retrospective studies and clinical trials (58–60). The use of targeted and immunotherapies has further expanded combination therapy strategies. A prospective study reported that for patients with gastric cancer-positive exfoliated cells (P0CY1), the success rate of conversion therapy was as high as 77.78% after treatment with NIPS combined with apatinib (61). Another study utilized local intraperitoneal infusion of chimeric antigen receptor T-cell (CAR-T) to effectively clear metastatic foci with high PD-L1 expression. This method’s property of blocking the immunosuppressive microenvironment and enhancing T-cell persistence provides a new strategy for immunotherapy of GCPM (62).The above study also emphasized the importance of conversion surgery after systemic therapy and intraperitoneal chemotherapy in improving prognosis. However, large-scale clinical trials are still needed to validate their standardized treatment protocols.

## 5 Limitations

There are several limitations of this study. First, only the WoSCC database was used for the literature search, which may overlook important literature in databases such as Scopus and PubMed, and by screening only English-language literature, high-quality non-English-language studies from East Asian countries such as Japan and China may have been missed. Future research needs to incorporate multilingual databases (e.g., J-STAGE, CNKI) to enhance global representation. Second, citation lag effects may underestimate the high quality of recently published articles. Furthermore, regional dominance may distort keyword trends and underestimate the contribution of other countries in GCPM research. Finally, for the literature screening, the timeframe was only up to October 31, 2024. This resulted in fewer publications in 2024 than before, affecting the accuracy of the analysis of the publication growth rate. Future research should consider expanding the range of languages and databases for literature screening and focusing on emerging research findings.

## 6 Conclusion

This study used bibliometric methods to conduct a comprehensive analysis of GCPM-related literature published between 2004 and 2024 to systematically summarize the global research landscape in this area. Japan, China, and the United States are the leading research forces, with Japanese institutions and researchers standing out in productivity and academic influence. Research focuses on four major directions: molecular mechanisms, prognostic evaluation systems, optimization of systemic chemotherapy regimens, and innovation of intraperitoneal therapies. Key pathways of GCPM, early diagnosis driven by AI and multi-omics, novel intraperitoneal therapeutic modalities, and immune/targeted drugs will become the hot spots of GCPM research. The focus of research is gradually shifting from early basic treatment to more precise and personalized diagnosis and treatment. Although systemic chemotherapy is still the cornerstone, introducing new therapeutic strategies drives the paradigm shift toward precision medicine. In the future, it is necessary to deepen the clinical translation guided by basic research, integrating advanced diagnostic techniques and multimodal treatment options to improve the prognosis of the GCPM population.

## Data Availability

The original contributions presented in the study are included in the article/supplementary material. Further inquiries can be directed to the corresponding authors.

## References

[1] Thrift AP El-Serag HB . Burden of gastric cancer. Clin Gastroenterol Hepatol. (2020) 18:534–42. doi: 10.1016/j.cgh.2019.07.045 PMC8859863 31362118

[2] Bray F Laversanne M Sung H Ferlay J Siegel RL Soerjomataram I . Global cancer statistics 2022: GLOBOCAN estimates of incidence and mortality worldwide for 36 cancers in 185 countries. CA Cancer J Clin. (2024) 74:229–63. doi: 10.3322/caac.21834 38572751

[3] Riihimäki M HEMMINKI A SUNDQUIST K SUNDQUIST J HEMMINKI K . Metastatic spread in patients with gastric cancer. Oncotarget. (2016) 7:52307–16. doi: 10.18632/oncotarget.10740 PMC5239553 27447571

[4] Verstegen MH Harker M Van De Water C Van Dieren J Hugen N Nagtegaal ID . Metastatic pattern in esophageal and gastric cancer: Influenced by site and histology. World J Gastroenterol. (2020) 26:6037–46. doi: 10.3748/wjg.v26.i39.6037 PMC7584055 33132653

[5] Thomassen I van Gestel YR van Ramshorst B Luyer MD Bosscha K Nienhuijs SW . Peritoneal carcinomatosis of gastric origin: a population-based study on incidence, survival and risk factors. Int J Cancer. (2014) 134:622–8. doi: 10.1002/ijc.v134.3 23832847

[6] Sirody J Kaji AH Hari DM Chen KT . Patterns of gastric cancer metastasis in the United States. Am J Surg. (2022) 224:445–8. doi: 10.1016/j.amjsurg.2022.01.024 35144812

[7] Kroese TE Takahashi Y Lordick F Van Rossum PSN Ruurda JP Lagarde SM . Liver oligometastatic disease in synchronous metastatic gastric cancer patients: a nationwide population-based cohort study. Eur J Cancer. (2023) 179:65–75. doi: 10.1016/j.ejca.2022.11.011 36509000

[8] Bando E Makuuchi R Irino T Tanizawa Y Kawamura T Terashima M . Validation of the prognostic impact of the new tumor-node-metastasis clinical staging in patients with gastric cancer. Gastric Cancer. (2019) 22:123–9. doi: 10.1007/s10120-018-0799-9 29357013

[9] Virgilio E Giarnieri E Giovagnoli MR Montagnini M Proietti A D’urso R . Gastric cancer cells in peritoneal lavage fluid: A systematic review comparing cytological with molecular detection for diagnosis of peritoneal metastases and prediction of peritoneal recurrences. Anticancer Res. (2018) 38:1255–62. 10.21873/anticanres.12347 29491048

[10] Guan WL He Y Xu RH . Gastric cancer treatment: recent progress and future perspectives. J Hematol Oncol. (2023) 16:57. doi: 10.1186/s13045-023-01451-3 37245017 PMC10225110

[11] Smyth EC Nilsson M Grabsch HI Van Grieken NC Lordick F . Gastric cancer. Lancet. (2020) 396:635–48. doi: 10.1016/S0140-6736(20)31288-5 32861308

[12] Wang Y Zhang L Yang Y Lu S Chen H . Progress of gastric cancer surgery in the era of precision medicine. Int J Biol Sci. (2021) 17:1041–9. doi: 10.7150/ijbs.56735 PMC8040314 33867827

[13] Thompson DF Walker CK . A descriptive and historical review of bibliometrics with applications to medical sciences. Pharmacotherapy. (2015) 35:551–9. doi: 10.1002/phar.2015.35.issue-6 25940769

[14] Pan X Yan E Cui M Hua W . Examining the usage, citation, and diffusion patterns of bibliometric mapping software: A comparative study of three tools. J informetrics. (2018) 12:481–93. doi: 10.1016/j.joi.2018.03.005

[15] Van Eck N Waltman L . Software survey: VOSviewer, a computer program for bibliometric mapping. scientometrics. (2010) 84:523–38. doi: 10.1007/s11192-009-0146-3 PMC2883932 20585380

[16] Bure IV Nemtsova MV Zaletaev DV . Roles of E-cadherin and noncoding RNAs in the epithelial-mesenchymal transition and progression in gastric cancer. Int J Mol Sci. (2019) 20(12):2870. doi: 10.3390/ijms20122870 31212809 PMC6627057

[17] Zhao JJ Ong CJ Srivastava S Chia DKA Ma H Huang K . Spatially resolved niche and tumor microenvironmental alterations in gastric cancer peritoneal metastases. Gastroenterology. (2024) 167:1384–1398.e4. doi: 10.1053/j.gastro.2024.08.007 39147169

[18] Cristescu R Lee J Nebozhyn M Kim KM Ting JC Wong SS . Molecular analysis of gastric cancer identifies subtypes associated with distinct clinical outcomes. Nat Med. (2015) 21:449–56. doi: 10.1038/nm.3850 25894828

[19] Tanaka Y Chiwaki F Kojima S Kawazu M Komatsu M Ueno T . Multi-omic profiling of peritoneal metastases in gastric cancer identifies molecular subtypes and therapeutic vulnerabilities. Nat Cancer. (2021) 2:962–77. doi: 10.1038/s43018-021-00240-6 35121863

[20] Miyamoto S Nagano Y Miyazaki M Nagamura Y Sasaki K Kawamura T . Integrin α5 mediates cancer cell-fibroblast adhesion and peritoneal dissemination of diffuse-type gastric carcinoma. Cancer Lett. (2022) 526:335–45. doi: 10.1016/j.canlet.2021.11.008 34775002

[21] Wang X Shi X Lu H Zhang C Li X Zhang T . Succinylation inhibits the enzymatic hydrolysis of the extracellular matrix protein fibrillin 1 and promotes gastric cancer progression. Adv Sci (Weinh). (2022) 9:e2200546. doi: 10.1002/advs.202200546 35901491 PMC9507347

[22] Lv ZD Na D Liu FN Du ZM Sun Z Li Z . Induction of gastric cancer cell adhesion through transforming growth factor-beta1-mediated peritoneal fibrosis. J Exp Clin Cancer Res. (2010) 29:139. doi: 10.1186/1756-9966-29-139 21034459 PMC2984409

[23] Wang C Ji J Jin Y Sun Y Cai Q Jiang J . Tumor-mesothelium HOXA11-PDGF BB/TGF β1-miR-181a-5p-Egr1 feedforward amplifier circuity propels mesothelial fibrosis and peritoneal metastasis of gastric cancer. Oncogene. (2024) 43:171–88. doi: 10.1038/s41388-023-02891-4 PMC10786717 37989866

[24] Wang R Song S Harada K Ghazanfari Amlashi F Badgwell B Pizzi MP . Multiplex profiling of peritoneal metastases from gastric adenocarcinoma identified novel targets and molecular subtypes that predict treatment response. Gut. (2020) 69:18–31. doi: 10.1136/gutjnl-2018-318070 31171626 PMC6943252

[25] Yasuda T Koiwa M Yonemura A Miyake K Kariya R Kubota S . Inflammation-driven senescence-associated secretory phenotype in cancer-associated fibroblasts enhances peritoneal dissemination. Cell Rep. (2021) 34:108779. doi: 10.1016/j.celrep.2021.108779 33626356

[26] Liu M Li H Zhang H Zhou H Jiao T Feng M . RBMS1 promotes gastric cancer metastasis through autocrine IL-6/JAK2/STAT3 signaling. Cell Death Dis. (2022) 13:287. doi: 10.1038/s41419-022-04747-3 35361764 PMC8971453

[27] Xie M Yu T Jing X Ma L Fan Y Yang F . Exosomal circSHKBP1 promotes gastric cancer progression via regulating the miR-582-3p/HUR/VEGF axis and suppressing HSP90 degradation. Mol Cancer. (2020) 19:112. doi: 10.1186/s12943-020-01208-3 32600329 PMC7322843

[28] Chen X Wu Z He Y Hao Z Wang Q Zhou K . Accurate and rapid detection of peritoneal metastasis from gastric cancer by AI-assisted stimulated raman molecular cytology. Adv Sci (Weinh). (2023) 10:e2300961. doi: 10.1002/advs.202300961 37114845 PMC10375130

[29] Dong D Tang L Li ZY Fang MJ Gao JB Shan XH . Development and validation of an individualized nomogram to identify occult peritoneal metastasis in patients with advanced gastric cancer. Ann Oncol. (2019) 30:431–8. doi: 10.1093/annonc/mdz001 PMC6442651 30689702

[30] Jiang Y Liang X Wang W Chen C Yuan Q Zhang X . Noninvasive prediction of occult peritoneal metastasis in gastric cancer using deep learning. JAMA Netw Open. (2021) 4:e2032269. doi: 10.1001/jamanetworkopen.2020.32269 33399858 PMC7786251

[31] Jiang Y Zhang Z Yuan Q Wang W Wang H Li T . Predicting peritoneal recurrence and disease-free survival from CT images in gastric cancer with multitask deep learning: a retrospective study. Lancet Digit Health. (2022) 4:e340–50. doi: 10.1016/S2589-7500(22)00040-1 35461691

[32] Ding P Yang J Guo H Wu J Wu H Li T . Multimodal artificial intelligence-based virtual biopsy for diagnosing abdominal lavage cytology-positive gastric cancer. Adv Sci (Weinh). (2025) 12(15):e2411490. doi: 10.1002/advs.202411490 39985379 PMC12005817

[33] Sun Y Chen Y Zhuang W Fang S Chen Q Lian M . Gastric cancer peritoneal metastasis related signature predicts prognosis and sensitivity to immunotherapy in gastric cancer. J Cell Mol Med. (2023) 27:3578–90. doi: 10.1111/jcmm.v27.22 PMC10660625 37605453

[34] Japanese gastric cancer treatment guidelines 2021 (6th edition). Gastric Cancer. (2023) 26:1–25. doi: 10.1007/s10120-022-01331-8 36342574 PMC9813208

[35] Wei YY Cai JY Wang LL Yang J Li YL Li XT . Dynamic change in the peritoneal cancer index based on CT after chemotherapy in the overall survival prediction of gastric cancer patients with peritoneal metastasis. J Cancer Res Clin Oncol. (2024) 150:222. doi: 10.1007/s00432-024-05707-4 38687350 PMC11061045

[36] Chen QY Liu ZY Zhong Q Jiang W Zhao YJ Li P . An intraoperative model for predicting survival and deciding therapeutic schedules: A comprehensive analysis of peritoneal metastasis in patients with advanced gastric cancer. Front Oncol. (2020) 10:550526. doi: 10.3389/fonc.2020.550526 33102217 PMC7546781

[37] Que SJ Chen QY Qing Z Liu ZY Wang JB Lin JX . Application of preoperative artificial neural network based on blood biomarkers and clinicopathological parameters for predicting long-term survival of patients with gastric cancer. World J Gastroenterol. (2019) 25:6451–64. doi: 10.3748/wjg.v25.i43.6451 PMC6881508 31798281

[38] Al-Batran SE Homann N Pauligk C Illerhaus G Martens UM Stoehlmacher J . Effect of neoadjuvant chemotherapy followed by surgical resection on survival in patients with limited metastatic gastric or gastroesophageal junction cancer: the AIO-FLOT3 trial. JAMA Oncol. (2017) 3:1237–44. doi: 10.1001/jamaoncol.2017.0515 PMC5824287 28448662

[39] Al-Batran SE Homann N Pauligk C Goetze TO Meiler J Kasper S . Perioperative chemotherapy with fluorouracil plus leucovorin, oxaliplatin, and docetaxel versus fluorouracil or capecitabine plus cisplatin and epirubicin for locally advanced, resectable gastric or gastro-oesophageal junction adenocarcinoma (FLOT4): a randomised, phase 2/3 trial. Lancet. (2019) 393:1948–57. doi: 10.1016/S0140-6736(18)32557-1 30982686

[40] Shitara K Bang YJ Iwasa S Sugimoto N Ryu MH Sakai D . Trastuzumab deruxtecan in previously treated HER2-positive gastric cancer. N Engl J Med. (2020) 382:2419–30. doi: 10.1056/NEJMoa2004413 32469182

[41] Fuchs CS Tomasek J Yong CJ Dumitru F Passalacqua R Goswami C . Ramucirumab monotherapy for previously treated advanced gastric or gastro-oesophageal junction adenocarcinoma (REGARD): an international, randomised, multicentre, placebo-controlled, phase 3 trial. Lancet. (2014) 383:31–9. doi: 10.1016/S0140-6736(13)61719-5 24094768

[42] Wilke H Muro K Van Cutsem E Oh SC Bodoky G Shimada Y . Ramucirumab plus paclitaxel versus placebo plus paclitaxel in patients with previously treated advanced gastric or gastro-oesophageal junction adenocarcinoma (RAINBOW): a double-blind, randomised phase 3 trial. Lancet Oncol. (2014) 15:1224–35. doi: 10.1016/S1470-2045(14)70420-6 25240821

[43] Li J Qin S Xu J Xiong J Wu C Bai Y . Randomized, double-blind, placebo-controlled phase III trial of apatinib in patients with chemotherapy-refractory advanced or metastatic adenocarcinoma of the stomach or gastroesophageal junction. J Clin Oncol. (2016) 34:1448–54. doi: 10.1200/JCO.2015.63.5995 26884585

[44] Shitara K Lordick F Bang YJ Enzinger P Ilson D Shah MA . Zolbetuximab plus mFOLFOX6 in patients with CLDN18.2-positive, HER2-negative, untreated, locally advanced unresectable or metastatic gastric or gastro-oesophageal junction adenocarcinoma (SPOTLIGHT): a multicentre, randomised, double-blind, phase 3 trial. Lancet. (2023) 401:1655–68. doi: 10.1016/S0140-6736(23)00620-7 37068504

[45] Janjigian YY Shitara K Moehler M Garrido M Salman P Shen L . First-line nivolumab plus chemotherapy versus chemotherapy alone for advanced gastric, gastro-oesophageal junction, and oesophageal adenocarcinoma (CheckMate 649): a randomised, open-label, phase 3 trial. Lancet. (2021) 398:27–40. doi: 10.1016/S0140-6736(21)00797-2 34102137 PMC8436782

[46] Fuchs CS Doi T Jang RW Muro K Satoh T MaChado M . Safety and efficacy of pembrolizumab monotherapy in patients with previously treated advanced gastric and gastroesophageal junction cancer: phase 2 clinical KEYNOTE-059 trial. JAMA Oncol. (2018) 4:e180013. doi: 10.1001/jamaoncol.2018.0013 29543932 PMC5885175

[47] Shitara K Rha SY Wyrwicz LS Oshima T Karaseva N Osipov M . Neoadjuvant and adjuvant pembrolizumab plus chemotherapy in locally advanced gastric or gastro-oesophageal cancer (KEYNOTE-585): an interim analysis of the multicentre, double-blind, randomised phase 3 study. Lancet Oncol. (2024) 25:212–24. doi: 10.1016/S1470-2045(23)00541-7 38134948

[48] André T TOUGERON D PIESSEN G De La Fouchardière C LOUVET C ADENIS A . Neoadjuvant nivolumab plus ipilimumab and adjuvant nivolumab in localized deficient mismatch repair/microsatellite instability-high gastric or esophagogastric junction adenocarcinoma: the GERCOR NEONIPIGA phase II study. J Clin Oncol. (2023) 41:255–65. 10.1200/JCO.22.00686 PMC9839243 35969830

[49] Shitara K Ajani JA Moehler M Garrido M Gallardo C Shen L . Nivolumab plus chemotherapy or ipilimumab in gastro-oesophageal cancer. Nature. (2022) 603:942–8. doi: 10.1038/s41586-022-04508-4 PMC8967713 35322232

[50] Bonnot PE Piessen G Kepenekian V Decullier E Pocard M Meunier B . Cytoreductive surgery with or without hyperthermic intraperitoneal chemotherapy for gastric cancer with peritoneal metastases (CYTO-CHIP study): A propensity score analysis. J Clin Oncol. (2019) 37:2028–40. doi: 10.1200/JCO.18.01688 31084544

[51] Rau B Lang H Koenigsrainer A Gockel I Rau HG Seeliger H . Effect of hyperthermic intraperitoneal chemotherapy on cytoreductive surgery in gastric cancer with synchronous peritoneal metastases: the phase III GASTRIPEC-I trial. J Clin Oncol. (2024) 42:146–56. doi: 10.1200/JCO.22.02867 PMC10824373 37906724

[52] Patel M Arora A Mukherjee D Mukherjee S . Effect of hyperthermic intraperitoneal chemotherapy on survival and recurrence rates in advanced gastric cancer: a systematic review and meta-analysis. Int J Surg. (2023) 109:2435–50. doi: 10.1097/JS9.0000000000000457 PMC10442139 37158149

[53] Kobayashi D Kodera Y . Intraperitoneal chemotherapy for gastric cancer with peritoneal metastasis. Gastric Cancer. (2017) 20:111–21. doi: 10.1007/s10120-016-0662-9 27803990

[54] Alyami M Bonnot PE Mercier F Laplace N Villeneuve L Passot G . Pressurized intraperitoneal aerosol chemotherapy (PIPAC) for unresectable peritoneal metastasis from gastric cancer. Eur J Surg Oncol. (2021) 47:123–7. doi: 10.1016/j.ejso.2020.05.021 32561204

[55] Case A Prosser S Peters CJ Adams R Gwynne S . Pressurised intraperitoneal aerosolised chemotherapy (PIPAC) for gastric cancer with peritoneal metastases: A systematic review by the PIPAC UK collaborative. Crit Rev Oncol Hematol. (2022) 180:103846. doi: 10.1016/j.critrevonc.2022.103846 36257535

[56] Sundar R Chia DKA Zhao JJ Lee A Kim G Tan HL . Phase I PIANO trial-PIPAC-oxaliplatin and systemic nivolumab combination for gastric cancer peritoneal metastases: clinical and translational outcomes. ESMO Open. (2024) 9:103681. doi: 10.1016/j.esmoop.2024.103681 39288528 PMC11421236

[57] Luksta M Bausys A Bickaite K Rackauskas R Paskonis M Luksaite-Lukste R . Pressurized intraperitoneal aerosol chemotherapy (PIPAC) with cisplatin and doxorubicin in combination with FOLFOX chemotherapy as a first-line treatment for gastric cancer patients with peritoneal metastases: single-arm phase II study. BMC Cancer. (2023) 23:1032. doi: 10.1186/s12885-023-11549-z 37875869 PMC10599063

[58] Yang ZY Yuan F Lu S Xu W Wu JW Xi WQ . Efficacy and safety of conversion therapy by intraperitoneal and intravenous paclitaxel plus oral S-1 in gastric cancer patients with peritoneal metastasis: A prospective phase II study. Front Oncol. (2022) 12:905922. doi: 10.3389/fonc.2022.905922 35795055 PMC9251062

[59] Shi M Yang Z Lu S Liu W Ni Z Yao X . Oxaliplatin plus S-1 with intraperitoneal paclitaxel for the treatment of Chinese advanced gastric cancer with peritoneal metastases. BMC Cancer. (2021) 21:1344. doi: 10.1186/s12885-021-09027-5 34922478 PMC8684127

[60] Yang Z Lu S Shi M Yuan H Wang Z Ni Z . Oncological outcomes of conversion therapy in gastric cancer patients with peritoneal metastasis: a large-scale retrospective cohort study. Gastric Cancer. (2024) 27:387–99. doi: 10.1007/s10120-023-01452-8 PMC10896904 38143257

[61] Ding P Yang P Tian Y Guo H Liu Y Zhang Z . Neoadjuvant intraperitoneal and systemic paclitaxel combined with apatinib and S-1 chemotherapy for conversion therapy in gastric cancer patients with positive exfoliative cytology: a prospective study. J Gastrointest Oncol. (2021) 12:1416–27. doi: 10.21037/jgo-21-375 PMC8421905 34532099

[62] Ma Q He X Zhang B Guo F Ou X Yang Q . A PD-L1-targeting chimeric switch receptor enhances efficacy of CAR-T cell for pleural and peritoneal metastasis. Signal Transduct Target Ther. (2022) 7:380. doi: 10.1038/s41392-022-01198-2 36402752 PMC9675732

